# A Systems View of the Differences between *APOE* ε4 Carriers and Non-carriers in Alzheimer’s Disease

**DOI:** 10.3389/fnagi.2016.00171

**Published:** 2016-07-12

**Authors:** Shan Jiang, Ling Tang, Na Zhao, Wanling Yang, Yu Qiu, Hong-Zhuan Chen

**Affiliations:** ^1^Department of Pharmacology, Institute of Medical Sciences, Shanghai Jiao Tong University School of Medicine, ShanghaiChina; ^2^Department of Paediatrics and Adolescent Medicine, Li Ka Shing Faculty of Medicine, The University of Hong Kong, PokfulamHong Kong

**Keywords:** Alzheimer’s disease, *APOE* ε4, *APOE* ε4 carriers and non-carriers, weighted gene co-expression network analysis (WGCNA), hub gene cluster

## Abstract

*APOE* ε4 is the strongest genetic risk factor for late-onset Alzheimer’s disease (AD) and accounts for 50–65% of late-onset AD. Late-onset AD patients carrying or not carrying *APOE* ε4 manifest many clinico-pathological distinctions. Thus, we applied a weighted gene co-expression network analysis to identify specific co-expression modules in AD based on *APOE* ε4 stratification. Two specific modules were identified in AD *APOE* ε4 carriers and one module was identified in non-carriers. The hub genes of one module of AD *APOE* ε4 carriers were *ISOC1, ENO3, GDF10, GNB3, XPO4*, *ACLY* and *MATN2*. The other module of AD *APOE* ε4 carriers consisted of 10 hub genes including *ANO3, ARPP21, HPCA, RASD2, PCP4* and *ADORA2A*. The module of AD *APOE* ε4 non-carriers consisted of 16 hub genes including *DUSP5, TNFRSF18, ZNF331, DNAJB5* and *RIN1*. The module of AD *APOE* ε4 carriers including *ISOC1* and *ENO3* and the module of non-carriers contained the most highly connected hub gene clusters. mRNA expression of the genes in the cluster of the *ISOC1* and *ENO3* module of carriers was shown to be correlated in a time-dependent manner under *APOE* ε4 treatment but not under *APOE* ε3 treatment. In contrast, mRNA expression of the genes in the cluster of non-carriers’ module was correlated under *APOE* ε3 treatment but not under *APOE* ε4 treatment. The modules of carriers demonstrated genetic bases and were mainly enriched in hereditary disorders and neurological diseases, energy metabolism-associated signaling and G protein-coupled receptor-associated pathways. The module including *ISOC1* and *ENO3* harbored two conserved promoter motifs in its hub gene cluster that could be regulated by common transcription factors and miRNAs. The module of non-carriers was mainly enriched in neurological, immunological and cardiovascular diseases and was correlated with Parkinson’s disease. These data demonstrate that AD in *APOE* ε4 carriers involves more genetic factors and particular biological processes, whereas AD in *APOE* ε4 non-carriers shares more common pathways with other types of diseases. The study reveals differential genetic bases and pathogenic and pathological processes between carriers and non-carriers, providing new insight into the mechanisms of the differences between *APOE* ε4 carriers and non-carriers in AD.

## Introduction

Alzheimer’s disease (AD) is one of the leading causes of dementia and is characterized by cognitive decline with distinctive brain pathology such as amyloid plaques and neurofibrillary tangles (Selkoe, 2003). Rare familial AD (FAD) is an early onset disease and is caused by several definite and specific genes, such as amyloid precursor protein (*APP*) and presenilin 1 and 2 (*PSEN1*, *PSEN2*) (Scheuner et al., 1996; Campion et al., 1999). In contrast, the etiology of the most common form of non-familial late-onset AD appears to be more complicated. Thus far, many genetic loci have been identified to be risk factors of late-onset AD through genome-wide association studies (GWAS) (Harold et al., 2009; Naj et al., 2011; Kauwe et al., 2014). Moreover, alterations in many molecules and biological processes have been recognized in AD (Mattson, 2004). A biological process may involve dozens and even hundreds of molecules, and a molecule may participate in multiple biological processes. It seems impractical to uncover the relationships among all of these molecules and biological processes one by one. Thus, the exploration of the pathogenic and pathological mechanisms of AD in a systematic view may be more appropriate.

*APOE* is the strongest susceptible gene of late-onset AD (Wijsman et al., 2011). *APOE* exists as three polymorphic alleles ε2, ε3, and ε4. Individuals with one or two copies of *APOE* ε4 have a higher risk of developing AD than the carriers of other isoforms (Corder et al., 1993). AD patients carrying or not carrying *APOE* ε4 manifest many clinico-pathological distinctions. Patients who are *APOE* ε4 carriers perform worse on memory tasks than non-carriers (Marra et al., 2004). Patients of *APOE* ε4 non-carriers exhibit impairments in naming, mental speed and executive function (van der Vlies et al., 2007; Wolk et al., 2010). A positron emission tomography study indicated different perfusion profiles in the brains of *APOE* ε4 carriers and non-carriers during a working memory task (Scarmeas et al., 2004). Moreover, AD *APOE* ε4 carrier’s display significantly reduced blood flow in the temporal and hippocampal areas (Suwa et al., 2015). In addition, *APOE* ε4 carriers have greater amyloid deposition and *APOE* ε4 can predict the atrophy rates across brain regions affected by AD (Jack et al., 2015; Hua et al., 2016). Moreover, previous studies have demonstrated that different drug responses are observed in *APOE* ε4 carriers and non-carriers. A neuroprotective agent facilitating brain noradrenergic and vasopressinergic activities have been shown to improve the Minimum Mental State Examination (MMSE) score in *APOE* ε4 carriers but not in non-carriers (Richard et al., 1997). A higher dose of bapineuzumab, an anti-β-amyloid peptide (Aβ) monoclonal antibody, is needed to decrease cerebrospinal fluid phospho-tau concentration in *APOE* ε4 non-carriers than in carriers (Salloway et al., 2014). All the evidence indicates that different pathogenic and pathologic processes are involved in the disease progression of AD patients with different *APOE* ε4 statuses. *APOE* ε4 has been shown to affect Aβ aggregation, promote neurofibrillary tangle formation and impair synaptic plasticity (Bu, 2009), which are all pathological hallmarks of AD. However, these harmful effects of *APOE* ε4 do not fully explain the clinico-pathological phenotypic distinctions between *APOE* ε4 carriers and non-carriers of AD patients. Furthermore, *APOE* ε4 only accounts for 50–65% of late-onset AD. Therefore, patient stratification based on *APOE* ε4 status can allow for the exploration of the underlying mechanisms of clinico-pathological distinctions between *APOE* ε4 carriers and non-carriers and may further help to elucidate the molecular mechanisms of AD which could be masked when combining *APOE* ε4 carriers and non-carriers together.

The transcriptome bridges the gap between genetic variation and the function of the molecules. Knowing its structure permits the exploration of the comprehensive function of molecules that could be influenced by genetic and non-genetic factors. With the availability of a transcriptome dataset (Webster et al., 2009), we analyzed the transcription profiles of AD patients based on *APOE* ε4 status by applying a systems biology approach, weighted gene co-expression network analysis (WGCNA) (Langfelder and Horvath, 2008). WGCNA allows for the identification of groups of genes (called modules) whose expression is highly correlated within a network. Then, specific co-expression modules can be obtained by comparing the modules identified in different sub-datasets. Using this method, we identified different co-expression modules and revealed different biological processes involved in AD *APOE* ε4 carriers and non-carriers.

## Materials and Methods

### Microarray Dataset

The microarray dataset was downloaded from Gene Expression Ominibus (GEO)^1^. The dataset, GSE15222, measured on GPL2700 using Illumina Sentrix Human Ref-8 Expression Bead Chip which covers 24354 probesets consists of human cerebral cortex samples of 188 controls and 176 patients with diagnosis of late-onset AD. The original expression data were rank-invariant by BeadStudio software available from Illumina and the Illumina custom error model was used. Rank-invariant-normalized expression data were log_10_ transformed, and missing data were encoded as missing, rather than as a zero level of expression. Chips with average detection scores less than 0.99 (5% of control chips, 8% of late-onset AD chips) were excluded from the analysis. Transcripts that were detected in less than 90% of the case or 90% of the control series were excluded from our study. Finally 8650 high quality transcripts were obtained (Webster et al., 2009). Of the 188 controls, 40 were *APOE* ε4 carriers and 148 were non-carriers. Of the 176 patients, 121 were *APOE* ε4 carriers and 55 were non-carriers.

### Sub-dataset-specific Co-expression Module Detection by WGCNA

The WGCNA package which provides a robust set of R functions was used to detect sub-dataset-specific modules (Langfelder and Horvath, 2008). GSE15222 was divided into four sub-datasets: patients carrying *APOE* ε4, patients not carrying *APOE* ε4, controls carrying *APOE* ε4 and controls not carrying *APOE* ε4. Outliers were filtered out using Euclidian distance as the similarity measure and average linkage as an agglomeration method. For each sub-group, subjects were clustered based on their dissimilarity, and any arrays with average inter-subject correlation less than 2 standard deviations below the mean were removed. This process was repeated until no arrays needed to be removed (Oldham et al., 2008). One outlier was detected in AD *APOE* ε4 non-carriers and was excluded before the construction. A demographic depiction of the subjects was performed to examine the similarity of sub-datasets. To construct the network, Pearson correlation coefficients were calculated for all possible gene pairs, and then the coefficients were powered by an exponent β in each sub-group stratified by *APOE* ε4 and phenotypes. A high β maintains high adjacencies but pushes lower adjacencies toward zero. An optimal exponent β can reduce the false positive rate within the network to the utmost extent (Zhang and Horvath, 2005). The dynamic tree-cutting algorithm was then used to identify modules after the summarization of similar patterns of connectivity among genes as topological overlap matrix (TOM) (Langfelder et al., 2008). Modules with similar module eigengenes (ME) were merged together. Unsupervised average linkage hierarchical clustering identified 18 modules in AD *APOE* ε4 carriers, 21 modules in AD *APOE* ε4 non-carriers, 10 modules in control *APOE* ε4 carriers and 8 modules in control *APOE* ε4 non-carriers. Next, consensus modules (AD *APOE* ε4 carriers and non-carriers, AD and control *APOE* ε4 carriers, and AD and control *APOE* ε4 non-carriers) were constructed with a similar procedure to that illustrated above. A detailed description of consensus module construction can be found at the WGCNA tutorial website^2^.

The modules constructed using the sub-dataset of AD *APOE* ε4 carriers were compared with the consensus modules of AD *APOE* ε4 carriers and non-carriers, and with the consensus modules of AD and control *APOE* ε4 carriers to detect specific modules of AD *APOE* ε4 carriers. The modules constructed using the sub-dataset of AD *APOE* ε4 non-carriers were compared with the consensus modules of AD *APOE* ε4 carriers and non-carriers, and with the consensus modules of AD and control *APOE* ε4 non-carriers to detect specific modules of AD *APOE* ε4 non-carriers. Then, the module preservation statistic *Z*_summary_ was utilized to further examine the sub-dataset-specificity of the modules (Langfelder et al., 2011). Unlike the cross-tabulation test, *Z*_summary_ takes into account both overlaps in module membership and the density and connectivity patterns of the modules. The following recommended significant thresholds for *Z*_summary_ were adopted: *Z*_summary_ <2 implies no evidence of module preservation, 2 < *Z*_summary_ < 10 implies weak to moderate evidence of module preservation, and *Z*_summary_ > 10 implies strong evidence for module preservation. Greater module preservation corresponds to lesser specificity of the module to the sub-dataset. Thus, a *Z*_summary_ less than 2 indicates strong evidence of specificity of the module, a *Z*_summary_ between 2 and 10 indicates modest evidence of specificity of the module and a *Z*_summary_ more than 10 indicates no evidence of specificity of the module. Module networks were graphically depicted using the program Cytoscape^3^.

Then random samplings with two different levels (50 and 75%) were performed with each level carried out five times. The criterion of replication is that more than 80% of genes within one of the modules (violet, dark magenta and light cyan) detected among the total samples should be included in one module detected in random samplings. Moreover, modules that were obtained from random samplings and whose genes are overlapped more than 80% with violet, dark magenta or light cyan modules should be AD *APOE* ε4 carrier or non-carrier-specific.

Hub genes were identified by WGCNA via measures of intramodular connectivity and module membership (Zhang and Horvath, 2005; Dewey et al., 2011; Langfelder et al., 2011; de Jong et al., 2012). Intramodular connectivity measures how a given gene is connected, or co-expressed, with the genes of a particular module. Module membership measures the membership of the *i*-th gene with respect to a given module. Hub genes tend to own high values of intramodular connectivity and module membership.

Module eigengene values adjusted for phenotype, age and sex were used to test differential expression of conserved modules between AD and control *APOE* ε4 carriers and between AD and control *APOE* ε4 non-carriers, and the values adjusted for *APOE* ε4 status, age and sex were used to test differential expressions of conserved modules between AD *APOE* ε4 carriers and non-carriers.

### Hub Gene Complete Graph Detection

R package *graph* was utilized to detect whether there was/were complete graph(s) comprised of hub genes in sub-dataset-specific modules.

### Primary Neuron Culture and Quantitative RT-PCR

Primary neurons were derived from the hippocampus of Sprague–Dawley (SD) rats at postnatal day 1 as previously described (Kam et al., 2010). Neurons were plated at a density of 2 × 10^6^ cells/well on poly-L-lysine-treated 6-well plate. The neurons were treated with recombinant human APOE ε4 or ε3 at a concentration of 5 μg/ml or without APOE treatment on DIV19 and collected for RNA extraction after 24, 48, and 72 h of treatment. Total RNA was extracted using a Total RNA kit 1 from Sigma (St. Louis, MO, USA). RNA was reverse transcribed to synthesize cDNA by using M-MLV reverse transcriptase (Invitrogen, Carlsbad, CA, USA). Real-time PCR was performed using SYBR Green (Roche Diagnostics, Basel, Switzerland). GAPDH was used as the normalization control. The relative mRNA levels were calculated by a comparative Cp value. The primers used were: ENO3: sense: AGCTGCTACCTAGGCAC TCT, antisense: GGTTCCGTCCAGCTCAATCA; GNB3: sense: TTTCACTGGCCACGAGTCAG, antisense: CTCTCGTGGGAG TAGGCTGT; XPO4: sense: GGAATTCAGCAGACGGGAGA, antisense: TAGTGTTTTGGAGGGAGAAAATTCC; GDF10: sense: AATCATCAAGGCTGCCCGAA, antisense: CTGGACC AGAACTCGTGCTT; ISOC1: sense: GCTGCACTAACAAAAC GCCA, antisense: TCATGGGACGGCAGGATAGA; ACLY: sense: GGTAAGCTGGTGCTTACGGA, antisense: TCTGGA TGGCTGAGGTGGTA; DUSP5: sense: GCCGACATTAGCTCC CACTT, antisense: GCCAAAGTTGGGAGAGACCA; TNFR SF18: sense: CACGTGTCCCCGAGATACC, antisense: GTCCC CCAGACGACACTTTT; DNAJB5: sense: CTCCTCACCGCA GCACC, antisense: TTCTCCTCAGCGTTGGGTTC; HILPDA: sense: GCCTGCACGATCTAGTGTGA, antisense: GCACTCC TCTGGATGGATGG; TBC1D8: sense: TGTATTCTCCCATAGC ATGTGGT, antisense: GTCTCCTCAGCGATCAGAGC; BDNF: sense: ACTGTCCTGCTACCGCAGTTG, antisense: GGGTCGC AGAACCGCTAAA; ST8SIA5: sense: ACTTCGTCTTCCGGTG CAAT, antisense: GGAAGTCGTCCAGCATGTACT; CHST12: sense: GGTCTCCTTCGCCAACTTCA, antisense: AGCATCC TCATCCAGGGTCT; ATF5: sense: GTGCCTAGGGTACAGGA GGA, antisense: GCAGAGGGGAGACCTAGACA; CSNK1D: sense: CACCTCACAGATTCCCGGTC, antisense: GCTCTTG GAGCCTGTCCATT; PLIN2: sense: ATTCGCCAGGAAGAAT GTGC, antisense: TGGCATGTAGTGTGGAGCTG.

### Functional Annotations of Sub-dataset-specific Modules

Functional annotations of sub-dataset-specific modules were obtained by applying ingenuity pathway analysis (IPA)^4^. IPA is a large manually curated database of published information on mammalian biology and diseases. It is widely used for high quality gene set enrichment analyses. Fisher’s exact tests were utilized to calculate the *P*-values for each functional annotation by comparing the number of genes from the module of interest that participated in the specified IPA term against the total number of genes from the term in the background set (Khatri et al., 2012).

### GWAS Dataset Preparation and Module-Based GWAS Signal Enrichment Test

The GWAS (Myers) was carried out in the same population as that of the microarray dataset we utilized, and the dataset was directly downloaded from the Myers laboratory^5^. For replication, the GWAS dataset from the Multi-Site Collaborative Study for Genotype-Phenotype Associations in Alzheimer’s Disease (GenADA) (Li et al., 2008) was downloaded from dbGaP^6^, the GWAS dataset from the Alzheimer’s Disease Neuroimaging Initiative (ADNI) (Mueller et al., 2005) was downloaded from the ADNI database^7^, and the dataset from the International Genomics of Alzheimer’s Project (IGAP) (Lambert et al., 2013) was downloaded from http://www.pasteur-lille.fr/en/recherche/u744/igap/igap_download.php.

We excluded single nucleotide polymorphisms (SNPs) with a missing genotype rate > 0.1, a minor allele frequency (MAF) < 0.01 and a Hardy–Weinberg equilibrium (HWE) < 0.001. Finally, after quality control, 364048, 431284, and 567199 SNPs from the Myers, GenADA and ADNI datasets, respectively, were included for *APOE* ε4 carriers, and 374149, 427678, and 566459 SNPs from the Myers, GenADA and ADNI datasets, respectively, were included for *APOE* ε4 non-carriers. GWAS dataset preparation and quality control procedures were performed using the software package PLINK^8^, release v 1.07 (Purcell et al., 2007). Processed data of IGAP were downloaded from the following link: http://www.pasteur-lille.fr/en/recherche/u744/igap/igap_download.php.

A previously reported procedure of GWAS signal enrichment test, called *i*-GSEA4GWAS, was employed (Zhang et al., 2010). First, the maximum -log(*P-*value) of the SNPs located between 20 kb upstream and downstream of a gene boundary was assigned to represent the gene, and the min *P-*value method, which does not excessively penalize large genes if causative associations are proportionally more common in smaller genes, has been widely used in systems biology (Baranzini et al., 2009; Jia et al., 2010). Instead of the commonly used phenotype label permutation, SNP label permutations were implemented to generate the distribution of the enrichment score (*ES*), and then the gene set’s significance proportion based enrichment score (*SPES*) was calculated based on genes’ rank using the following equation: *SPES* = *k/K* ×*ES*, where *k* is the proportion of significant genes of the gene set and *K* is the proportion of significant genes of the total number of genes in the GWAS (Zhang et al., 2010). Gene-length bias was eliminated by applying adaptive permutation in PLINK before *i*-GSEA4GWAS. False discovery rate was applied for multiple comparison correction. Then, a comparative quantile-quantile (QQ) plot was used to demonstrate the differences among all genes and genes within the interested modules of *APOE* ε4 carriers, and the genomic dispersion factor, λ, was used to assess the strengths of genetic association signals and to quantify the differences when compared with the signals of all genes. The comparative QQ plot was produced by plotting the ranked -log_10_(*P*-value) against the expected order statistic, -log_10_[*i*/(*L*+1)], where *i* is the rank for each SNP *P*-value (1 = smallest and *L* = largest), and *L* is the number of SNPs. Function estlambda() within the R package GenABEL was utilized to estimate the genomic dispersion factor λ. If a co-expression module was genetically associated, significant SNPs within 20 kb upstream and downstream of the gene boundaries within the module in the sub-group in which it belongs (*APOE* ε4 carrier sub-group or *APOE* ε4 non-carrier sub-group) were identified, and the significance of these SNPs in its opposite sub-group were also calculated.

### Phylogenetically Conserved Promoter Motif Identification in Hub Genes of the Complete Graph

The conserved promoter motif(s) was/were identified by PhyloCon. PhyloCon, which stands for Phylogenetic Consensus, is one of the first motif-finding algorithms to combine the power of phylogenetic conservation and gene co-regulation (Wang and Stormo, 2003; Wang, 2007). PhyloCon first aligns conserved regions of orthologous sequences into multiple sequence alignments, or profiles, and then compares profiles representing non-orthologous sequences. Then, motifs represented by matrices or IUPAC strings emerge as common regions in these profiles (Wang, 2007). An online web server named WebLogo was used to generate the sequence logos of the motifs (Crooks et al., 2004). In the present study, orthologous sequences of the species *Homo sapiens, Rattus norvegicus, Mus musculus*, and *Canis lupus familiaris* were utilized. For promoter regions, the 4 kb segments centered on the annotated transcription start site (TSS) of each human RefSeq gene were extracted according to a previous study’s method (Xie et al., 2005). If the annotated translation start codon was within 2 kb of the TSS, the shorter region that did not overlap the protein-coding sequence was selected. For genes with alternatively spliced first exons, all promoters were included. Details for promoter region detection are given in a previous study (Xie et al., 2005).

JASPAR CORE database and miRWalk were utilized to identify common human transcription factors (TFs), TF binding sites (TFBS) and common miRNAs of the conserved motif(s) and these highly connected genes. The JASPAR CORE database, which contains a curated, non-redundant set of profiles, is derived from published collections of experimentally defined transcription factor binding sites for eukaryotes, The matrices and IUPAC strings were then input into the JASPAR CORE database to identify common human TFs and the TF binding sites (TFBS) of these co-expressed genes (Mathelier et al., 2014). miRWalk, a comprehensive database that incorporates miRNA-targets interactions information produced by eight established miRNA prediction programs on 3′ UTRs of all known genes, was utilized to evaluate whether some miRNAs exist as the common regulators of the mRNAs of genes within the same complete graph post-transcriptionally (Dweep et al., 2011). In the present study, five default prediction programs, miRanda, miRDB, miRWalk, RNA22, and TargetScan, were used. If a miRNA was predicted by at least two prediction programs, it was regarded to bind to the gene with high probability.

### AD and Parkinson’s Disease (PD), Bipolar Disease (BD) Consensus Pathogenic Module Identification

GEO dataset GSE20295^9^ for PD and GSE12654^10^ for BD were extracted. The procedures for PD and BD co-expression network construction and module detection were the same as those of AD. Then, each PD- or BD-specific module was compared with AD-specific modules via hypergeometric tests which examined whether the number of genes overlapped between PD or BD and AD was significantly larger than that expected by chance.

## Results

### Specific Co-expression Module Detection in AD *APOE* ε4 Carriers and Non-carriers

**FIGURE 1:**
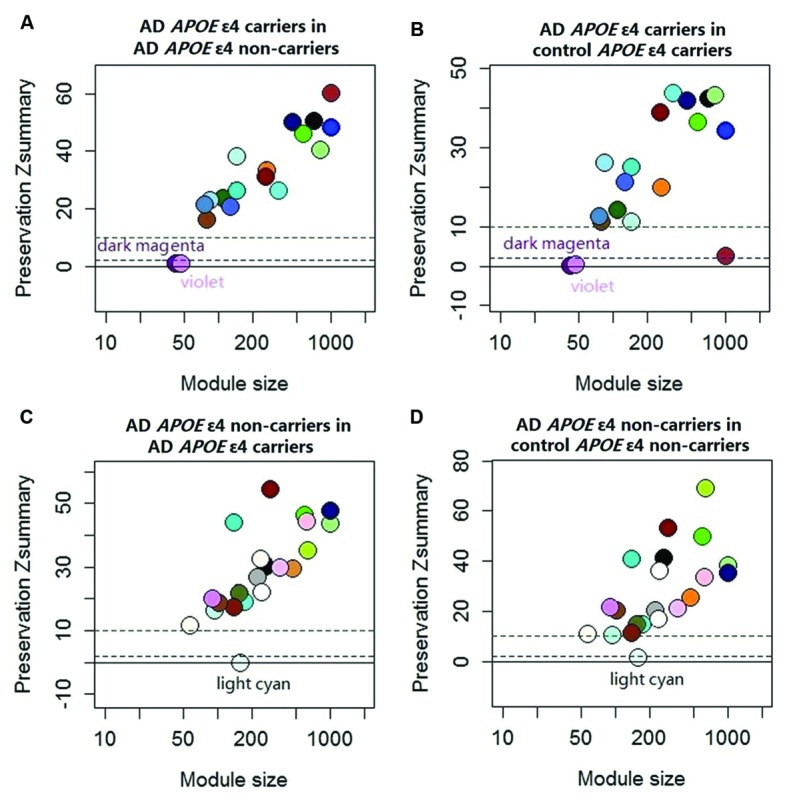
**Preservation statistic of modules detected in the test sub-dataset in the reference sub-dataset.** The *Z*_summary_ = 2 and *Z*_summary_ = 10 thresholds are indicated by dashed lines. *Z*_summary_ less than 2 implies no evidence of module preservation, which indicates strong evidence of module specificity. *Z*_summary_ between 2 and 10 implies modest evidence of module preservation and *Z*_summary_ more than 10 implies strong evidence of module preservation, which indicates modest or no evidence of module specificity. **(A)** Module preservation of AD *APOE* ε4 carriers in AD *APOE* ε4 non-carriers; **(B)** Module preservation of AD *APOE* ε4 carriers in control *APOE* ε4 carriers; **(C)** Module preservation of AD *APOE* ε4 non-carriers in AD *APOE* ε4 carriers; **(D)** module preservation of AD *APOE* ε4 non-carriers in control *APOE* ε4 non-carriers. See Supplementary Table S2 for exact *Z*_summary_ statistics.

**FIGURE 2:**
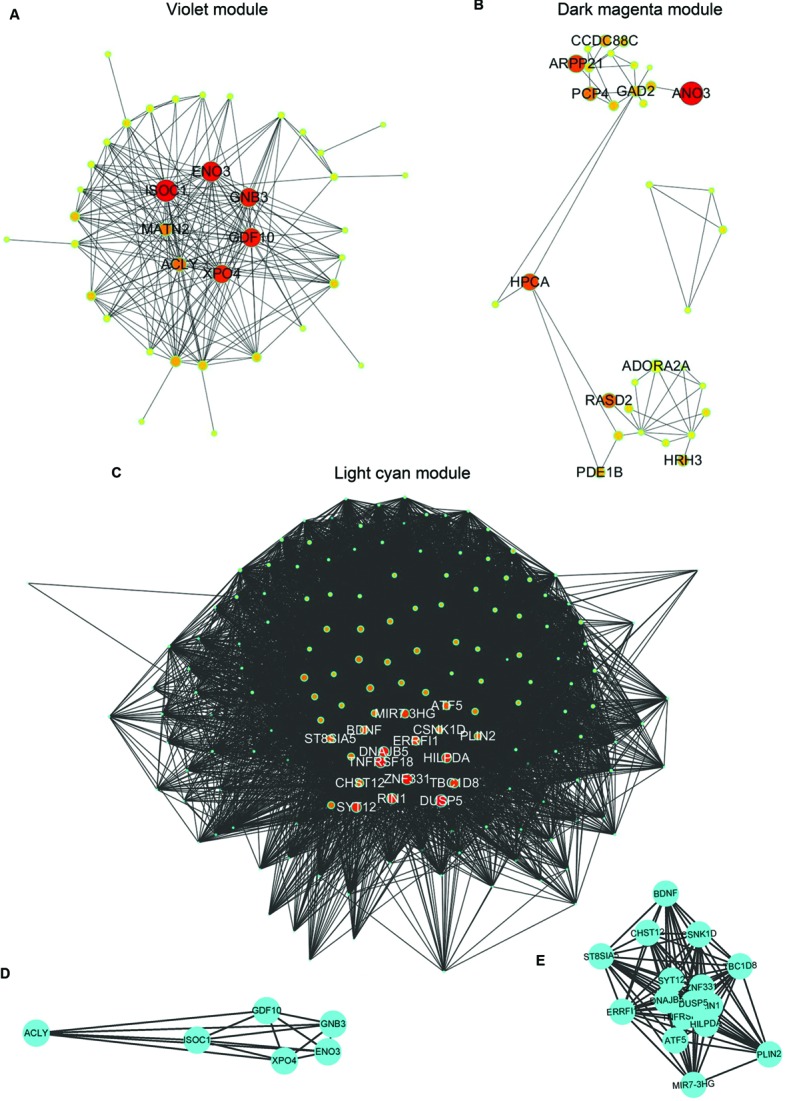
**Graphic visualization of modules and complete graphs identified in AD *APOE* ε4 carriers and non-carriers. (A–C)** Graphic visualization of the violet **(A)**, and dark magenta **(B)** modules for AD *APOE* ε4 carriers and the light cyan module **(C)** for AD *APOE* ε4 non-carriers with hub genes. Nodes were filled with different intermediate colors varying from red to ivory white, where red represents high intramodular connectivity and ivory white represents low connectivity. Edges were weighted by the strengths of the connections between two nodes, for which shorter edges correspond to stronger relationships and vice versa. **(D)** Complete graph of the top six hub genes in the violet module of AD *APOE* ε4 carriers. **(E)** Complete graph of the top sixteen hub genes in the light cyan module of AD *APOE* ε4 non-carriers.

The demographic characteristics of the enrolled subjects after excluding outlier samples demonstrated no differences (Supplementary Table S1). By applying WGCNA, we identified two specific co-expression modules (violet and dark magenta) in AD *APOE* ε4 carriers and one module (light cyan) in AD *APOE* ε4 non-carriers (**Figure 1**). The permutation-based preservation statistic, *Z*_summary_, demonstrated that the *Z*_summary_ statistics of the identified three modules were all less than 2 when assessing the violet and dark magenta modules of AD *APOE* ε4 carriers in AD *APOE* ε4 non-carriers and control *APOE* ε4 carriers and when assessing the light cyan module of AD *APOE* ε4 non-carriers in AD *APOE* ε4 carriers and control *APOE* ε4 non-carriers, showing no evidence of module preservation in these sub-datasets (**Figure 1**; Supplementary Table S2). These results strongly indicated that the three co-expression modules were sub-dataset-specific. Network visualizations of the violet and dark magenta modules in AD *APOE* ε4 carriers and the light cyan module in AD *APOE* ε4 non-carriers are shown in **Figures 2A–C**, respectively (Supplementary Tables S3–S5 for full list of genes of violet, dark magenta and light cyan modules respectively). The identified hub genes (most centered genes) are shown in Supplementary Tables S3–S5. Moreover, six of the seven hub genes (*ISOC1, ENO3, GDF10, GNB3, XPO4* and *ACLY*, Supplementary Table S3) of the violet module and all the hub genes of the light cyan module (Supplementary Table S5) were found to be highly connected as demonstrated to form a complete graph respectively, which represents the most highly connected gene cluster in biological network and tends to be very important (Jeong et al., 2001; Horvath and Dong, 2008) (**Figure 2D**; Supplementary Table S6 for the pairwise Pearson correlation coefficients for the violet module and **Figure 2E**; Supplementary Table S7 for the pairwise Pearson correlation coefficients for the light cyan module).

Furthermore, we examined whether the non-specific co-expression modules were differentially expressed between AD and control *APOE* ε4 carriers, between AD and control *APOE* ε4 non-carriers, and between AD *APOE* ε4 carriers and non-carriers. In the results, no differences were found (data not shown), demonstrating that these non-specific co-expression modules were completely conserved.

Due to the unavailability of other AD-associated datasets with *APOE*ε4 status, we carried out random samplings for replication. All three modules could be replicated (Supplementary Table S8).

### Highly Connected Hub Genes Detected in the Violet and Light Cyan Modules Co-expressed in a Time-Dependent Manner Dependent on APOE Types

**FIGURE 3:**
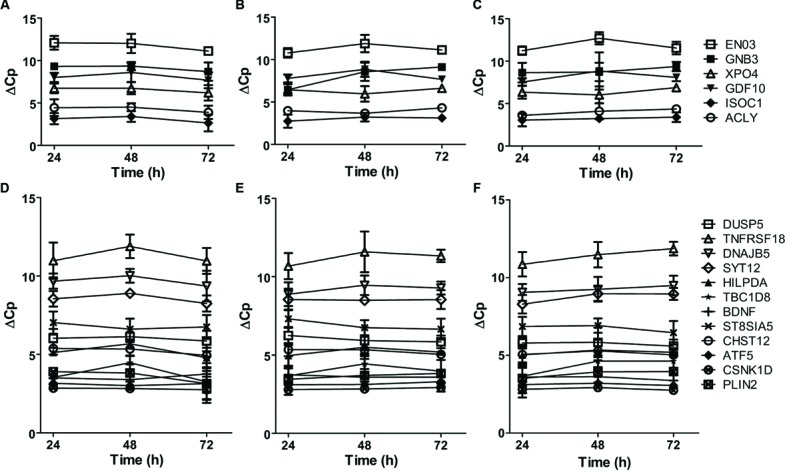
**Time-dependent mRNA expression of genes within the complete graphs of the violet and light cyan modules. (A–C)** The mRNA expression of six genes within the complete graph of the violet module of AD *APOE* ε4 carriers. The legends of the six genes are shown on the right. **(D–F)** The mRNA expression of twelve genes within the complete graph of the light cyan module of AD *APOE* ε4 non-carriers. The legends of the 12 genes are shown on the right. The primary cultured neurons were treated with recombinant human APOE ε4 **(A,D)** or ε3 **(B,E)** at the concentrations of 5 μg/ml or without APOE treatment **(C,F)** on DIV19 and collected for quantitative RT-PCR after 24, 48, and 72 h of treatment. The relative mRNA levels were calculated by a comparative Cp value. The data are presented as the mean ± SD from at least three independent experiments.

The highly connected hub genes in the complete graph implicate that the expression of these genes is correlated. Thus, we examined the co-expression patterns of these genes in primary cultured neurons treated with APOE ε3 or APOE ε4. As shown in **Figure 3A**, the mRNA expressions of all six genes (*ISOC1, ENO3, GDF10, GNB3, XPO4* and *ACLY*) in the complete graph of the violet module of AD *APOE* ε4 carriers demonstrated a similar time-dependent patterns in neurons treated with physiological concentration of APOE ε4, whereas, no such patterns were observed in neurons treated with APOE ε3 or in untreated neurons (**Figures 3B,C**). The pairwise Pearson correlation coefficient matrix of the expressions showed the high correlations between either two genes expressed in the APOE ε4-treated neurons (Supplementary Table S9) with a mean absolute Pearson correlation coefficient of 0.902, which is markedly higher than the mean absolute Pearson correlation coefficients for neurons treated with APOE ε3 (0.613) or for untreated neurons (0.571) (Supplementary Tables S10 and S11 for the pairwise Pearson correlation coefficient matrices for APOE ε3-treated neurons and for untreated neurons, respectively). These results confirm the co-expression of these six genes detected in the violet module.

The mRNA expression of 12 genes in the complete graph of light cyan module of AD *APOE* ε4 non-carriers displayed similar or opposite time-dependent patterns in primary cultured neurons treated with APOE ε3 (**Figure 3E**). The pairwise Pearson correlation coefficient matrix showed positive or negative correlations between most gene pairs (Supplementary Table S12), with a mean absolute Pearson correlation coefficient of 0.834. These results validated the co-expression of genes in the complete graph of the light cyan module. The co-expression patterns were much weaker in neurons treated with APOE ε4 and in untreated neurons (**Figure 3D**, Supplementary Table S13 for the pairwise Pearson correlation coefficient matrix for APOE ε4-treated neurons and **Figure 3F**, Supplementary Table S14 for untreated neurons, the mean Pearson correlation coefficients were 0.616 and 0.629, respectively).

### Differential Biological Processes Enriched in Specific Modules

Co-expressed genes in a module may directly interact with each other or take part in the same biological processes and signaling pathways. Hub genes within each module may play pivotal roles in module function. Thus, IPA was employed for further analysis.

**FIGURE 4:**
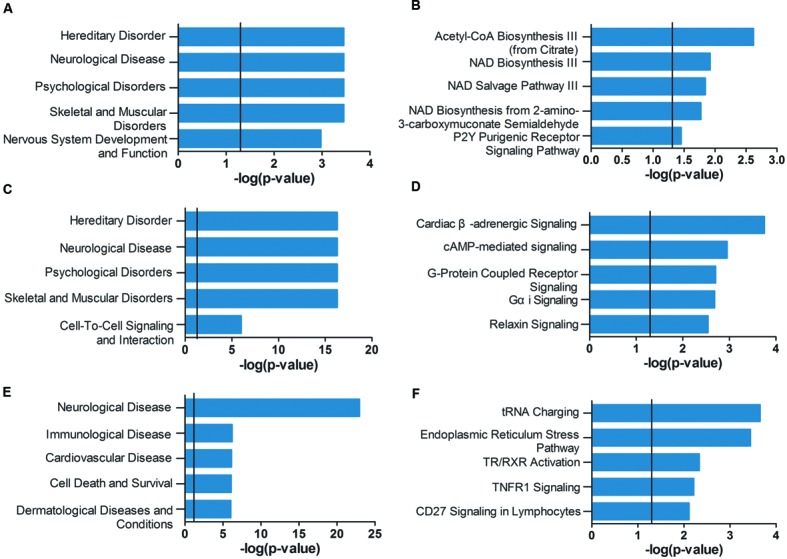
**Ingenuity pathway analysis (IPA) gene set enrichment analysis of the modules identified in AD *APOE* ε4 carriers and non-carriers.** IPA-identified top five diseases and functions **(A,C,E)** and top five canonical pathways **(B,D,F)** of the violet **(A,B)** and dark magenta **(C,D)** modules of AD *APOE* ε4 carriers and of the light cyan module **(E,F)** of AD *APOE* ε4 non-carriers. The verticals indicate the significant thresholds set by IPA.

As shown in **Figure 4A**, the genes of the violet module identified in AD *APOE* ε4 carriers were enriched in hereditary disorders, neurological diseases, psychological disorders, and nervous system development and function (Supplementary Table S15). The first two hub genes, *ISOC1* and *ENO3*, were enriched in the first annotation, hereditary disorders. These two hub genes and another hub gene *GNB3* were enriched in neurological diseases (Supplementary Table S16). The primarily enriched signaling pathways were acetyl-CoA biosynthesis III (from citrate), NAD biosynthesis III, NAD salvage pathway III and NAD biosynthesis from 2-amino-3-carboxymuconate semialdehyde (**Figure 4B**; Supplementary Table S17), which are mainly energy metabolism-associated signaling pathways. Two relatively important energy metabolism-associated signaling pathways, gluconeogenesis I and glycolysis I, were enriched by the hub gene *ENO3* (Supplementary Table S17). Genes of the dark magenta module were mostly enriched in hereditary disorders, neurological diseases, psychological disorders, and G protein-coupled receptors (GPCRs) and second messenger-associated signaling pathways of GPCRs (**Figures 4C,D**, see Supplementary Tables S18–S20 for all enriched IPA terms). The protein level of the hub gene, *GAD2*, which catalyzes glutamate to γ-aminobutyric acid, has been reported to be reduced in some cerebral regions of AD patients (Schwab et al., 2013).

Unlike the modules identified in AD *APOE* ε4 carriers, genes of the light cyan module identified in AD *APOE* ε4 non-carriers were enriched in some other types of diseases, such as immunological and cardiovascular diseases, in addition to the primarily enriched neurological diseases (**Figure 4E**, see also Supplementary Table S21). The top-ranked hub gene, *DUSP5*, was enriched in neurological diseases (Supplementary Table S22). Moreover, another hub gene, *BDNF*, has been widely reported to be related to AD (Tapia-Arancibia et al., 2008). Our analysis also demonstrated that approximately 66% of enriched genes found in either immunological or cardiovascular diseases were also found in neurological diseases (Supplementary Figure S1). However, genes within the two modules of AD *APOE* ε4 carriers were not highly enriched in immunological and cardiovascular diseases (Supplementary Tables S16 and S19). In addition, genes of the light cyan module were mainly enriched in the following signaling pathways: tRNA charging, endoplasmic reticulum (ER) stress pathway, TR/RXR (thyroid hormone receptor/retinoid X receptor) activation and TNFR1 (tumor necrosis factor receptor type 1) signaling (**Figure 4F**; Supplementary Table S23).

### Module-Based Enrichment of Genetic Association Signals

**FIGURE 5:**
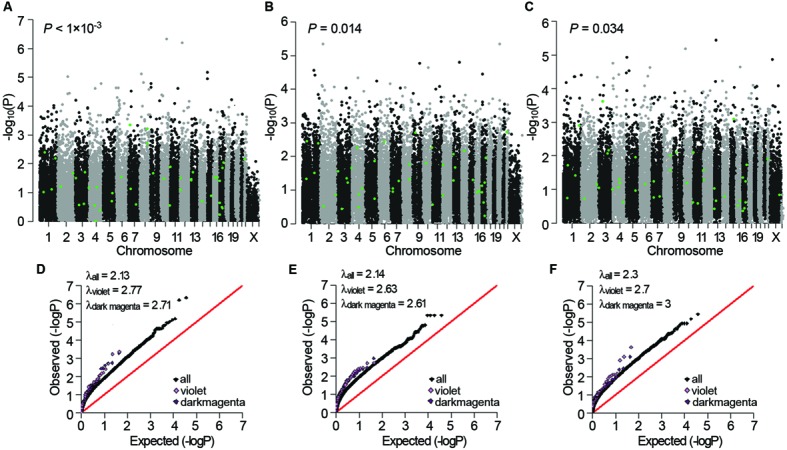
**Genetic association signals for the violet and dark magenta modules of AD *APOE* ε4 carriers. (A–C)** Manhattan plots of the enrichment analyses of the genetic association signals from the Myers GWAS **(A)**, GenADA GWAS **(B)**, and ADNI GWAS **(C)** datasets for the violet module of AD *APOE* ε4 carriers. The green dots represent genes within the violet module. **(D–F)** Comparative QQ plots of the genetic association signals from the Myers GWAS **(D)**, GenADA GWAS **(E)** and ADNI GWAS **(F)** datasets for the violet and dark magenta module of AD *APOE* ε4 carriers. The black dots represent all genes, the violet dots represent genes within the violet module and the dark magenta dots represent genes within the dark magenta module. Values of the genomic dispersion factor λ are given in the top-left of each comparative QQ plot.

Next, we examined whether the three sub-dataset-specific co-expression modules had genetic bases. The violet module in AD *APOE* ε4 carriers showed significant enrichment of signals from the GWAS dataset of Myers of the corresponding *APOE* ε4 carriers in the gene expression data [*P* < 0.001 and *P* < 0.001 after false discovery rate (FDR) correction, **Figure 5A**]. Moreover, the significant enrichment of signals was replicated in the other two GWAS datasets of *APOE* ε4 carriers (*P* = 0.014 for the data from GenADA and *P* = 0.034 for the data from ADNI, **Figures 5B,C**). The dark magenta module of AD *APOE* ε4 carriers only showed a significant enrichment of signals from the GenADA GWAS dataset of *APOE* ε4 carriers (*P* = 0.015, Supplementary Figure S2). Comparative QQ plots showed that genes within violet and dark magenta modules, especially the violet module, deviated from the expected values even further compared with the black dots, which represent all genes (**Figures 5D–F**). The larger genomic dispersion factors (λ_violet_ and λ_darkmagenta_) further validated their genetic bases (**Figures 5D–F**). Furthermore, genes in these two modules showed significant enrichment of signals from the IGAP GWAS dataset, which is the biggest AD-associated GWAS dataset to date (FDR-corrected *P-*value < 1 × 10^-4^ for the violet module and *P-*value = 0.009 for the dark magenta module, Supplementary Figure S3). The QQ plots also showed a deviation of genes from the expected values (Supplementary Figure S4). Moreover, almost all significant SNPs within the 20 kb gene boundaries of the violet module in *APOE* ε4 carriers were insignificant in non-carriers (Supplementary Table S24). The light cyan module in AD *APOE* ε4 non-carriers did not show a significant enrichment of signals.

### Analysis of Possible Transcription Modulation of Complete Graphs in Specific Modules

**FIGURE 6:**
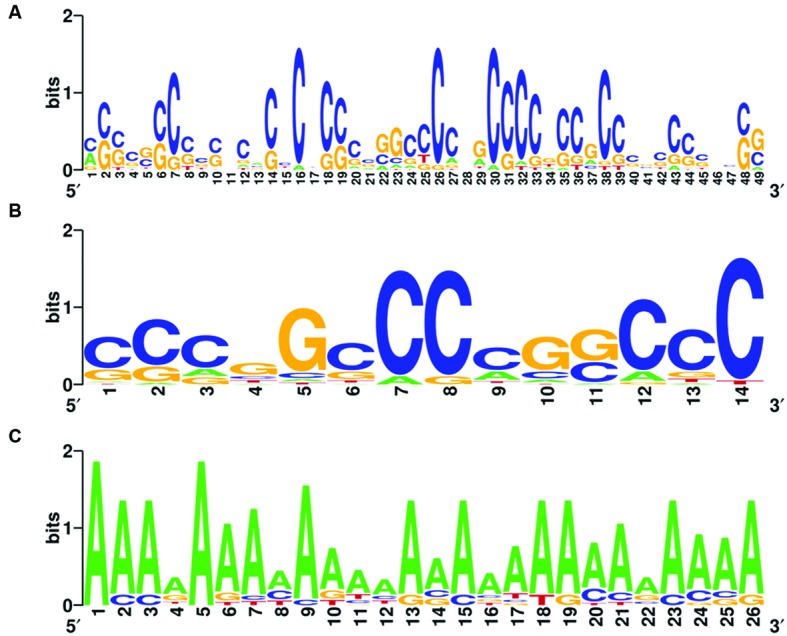
**Conserved promoter motifs of genes within the complete graphs of the violet and light cyan modules.** WebLogo representations of conserved promoter motifs in the violet module **(A,B)** of AD *APOE* ε4 carriers and in the light cyan module **(C)** of AD *APOE* ε4 non-carriers. The height of a letter at a particular position is proportional to the frequency of that nucleotide substitution in that position.

Complete graphs were detected and validated in the violet module of AD *APOE* ε4 carriers and in the light cyan module of AD *APOE* ε4 non-carriers, suggesting common transcription modulation. Thus, we first identified conserved promoter motif(s) by employing promoter homologous sequences of these genes from four species, *Homo sapiens, Rattus norvegicus, Mus musculus*, and *Canis lupus familiaris*. Two conserved promoter motifs with extremely low conservative *P*-values (*P* = 6.539 × 10^-122^ for the 49-base motif and *P* = 1.003 × 10^-74^ for the 14-base motif) were detected for the genes of the complete graph of the violet module of AD *APOE* ε4 carriers (**Figures 6A,B**), and an adenine-rich conserved promoter motif was identified with a *P*-value of 1.935 × 10^-125^ among the genes of the complete graph of the light cyan module of AD *APOE* ε4 non-carriers (**Figure 6C**).

Then, common human TFs of two motifs in the violet module were identified (**Table 1**). Interestingly, two TFs, ZNF263 and ESR1, were shared by the two motifs, and SNPs within *ESR1* have been widely reported to be associated with AD (Mattila et al., 2000; Corbo et al., 2006). RREB1, a TF of 49-base motif, which can potentiate the transcriptional activity of NeuroD1/β2 (Ray et al., 2003), has been reported to be related with AD and potentially with the *NFκB1* gene (Pasluosta et al., 2011). Another neuro-associated TF of 49-base motif, named REST, which can repress neuronal genes in non-neuronal tissues (Yeo et al., 2005), has been demonstrated to both silence and repress neuronal genes (Greenway et al., 2007). SNPs proximal to *TP63*, a TF of 14-base motif, which is present in the aging human hippocampus (Yang and Wang, 1994), are reported to be associated with brain morphometric measures of AD (Shen et al., 2010). Another TF of 14-base motif, named EGR1, up-regulates the *PSEN2* gene in neuronal cells (Renbaum et al., 2003). However, currently no highly scored human TFs could be predicted for the motif identified in the light cyan module of AD *APOE* ε4 non-carriers.

**Table 1:** Human TFs predicted for the two conserved promoter motifs in the violet module.

Motif	TF name	Class	Family	Score	Percent score
49-base motif	ZNF263	Zinc-coordinating	ββα-zinc finger	36.811	87.646
	RREB1	Zinc-coordinating	ββα-zinc finger	33.664	84.161
	ESR1	Zinc-coordinating	Hormone-nuclear receptor	33.618	84.045
	REST	Zinc-coordinating	ββα-zinc finger	32.833	78.173
	PAX5	Helix-turn-helix	Homeo	32.654	85.931
14-base motif	SP2	Zinc-coordinating	ββα-zinc finger	24.495	87.483
	ESR1	Zinc-coordinating	ββα-zinc finger	23.501	83.933
	TP63	Zinc-coordinating	Loop-sheet-helix	23.084	82.443
	ZNF263	Zinc-coordinating	ββα-zinc finger	23.082	82.435
	EGR1	Zinc-coordinating	ββα-zinc finger	22.879	81.711

*Top five predicted human TFs of the 49-base motif and 14-base motif from complete graph of violet module of AD *APOE* ε4 carriers.*

Furthermore, we investigated whether the mRNAs of these genes in the complete graph could be regulated by some common miRNAs. For the six genes in the violet module of AD *APOE* ε4 carriers, hsa-miR-194, hsa-miR-199a-5p, hsa-miR-199b-5p, hsa-miR-30a, hsa-miR-30d, and hsa-miR-30e were indicated to bind with at least four of the five hub genes except *GNB3* (Supplementary Table S25). hsa-miR-194 has been reported to be down-regulated in white matter of AD patients and to be negatively associated with neurofibrillary tangles in gray matter and in neuritic plaques and neurofibrillary tangles in white matter of AD patients (Wang et al., 2011). miRNAs in the hsa-miR-199 family have been shown to target genes involved in neurodegenerative diseases (Roshan et al., 2009). Five members of the miR-30 family are up-regulated in AD patients (Leidinger et al., 2013). However, currently no highly scored common miRNAs were predicted for the genes in the complete graph of the light cyan module of AD *APOE* ε4 non-carriers.

### Correlated Modules between AD and PD

Parkinson’s disease is also a neurodegenerative disorder with Lewy bodies deposited in neurons. Approximately 41.3% of PD patients are complicated with dementia according to a large population-based investigation (Mayeux et al., 1992). Pathologically, many PD patients show senile plaques and fibrillary tangles within the cerebral cortex (Boller et al., 1980), and many AD patients display Lewy bodies in cortical and subcortical regions (Hansen et al., 1990). BD is associated with an increased risk of dementia (Wu et al., 2013). Some symptoms and neuropathology of AD and BD overlap, such as brain atrophy, cognitive impairment, and emotional disturbances (Rao et al., 2012), however, no plaques and fibrillary tangles have been reported to be characteristic of BD. Thus, to further validate the identified modules, we applied the WGCNA analysis to these two neurological diseases to investigate whether the three identified co-expression modules were related to these diseases.

**FIGURE 7:**
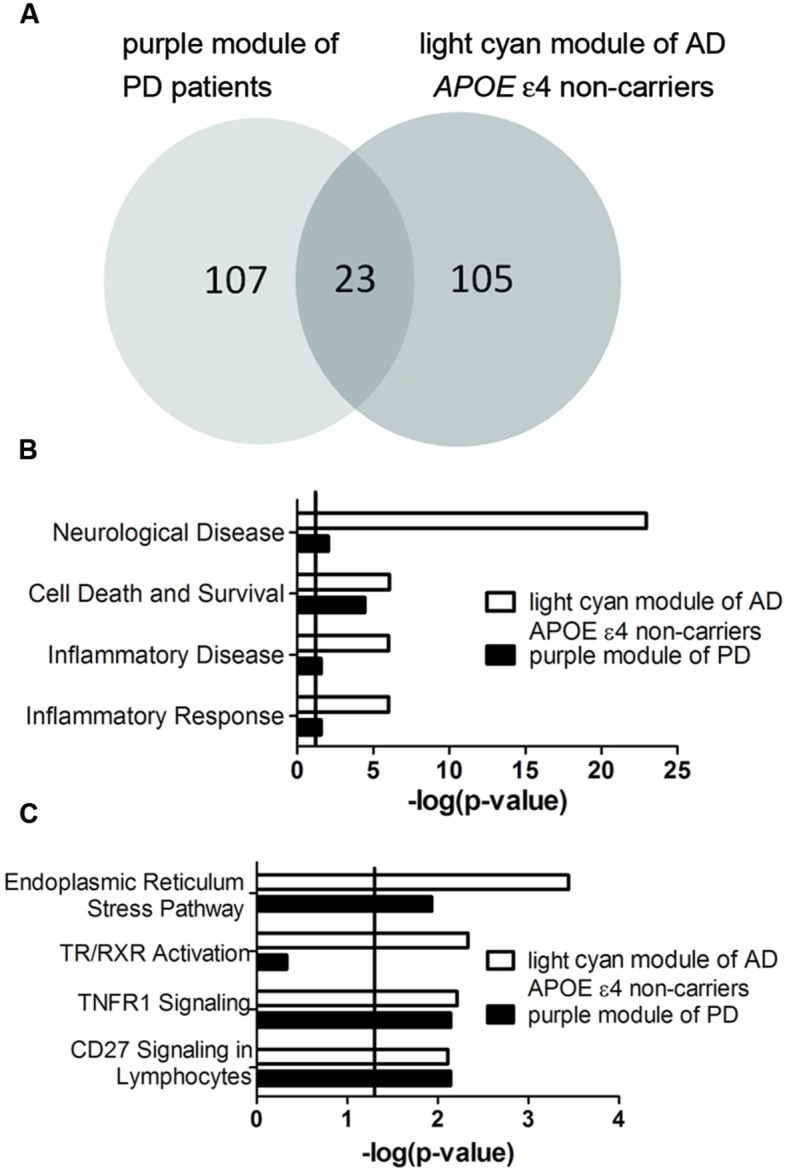
**Correlation between purple module of PD patients and light cyan module of AD *APOE* ε4 non-carriers. (A)** The overlapped gene numbers between these two modules shown in a venn diagram. **(B,C)** The top-shared diseases and functions **(B)** and canonical pathways **(C)** between the two modules. The verticals indicate the significant thresholds set by IPA.

Transcriptomic data from three brain regions– Brodmann area 9 (BA9) of the prefrontal cortex, the putamen (PT) and the entire substantia nigra (SN) of PD patients were available for analysis. Among the detected PD-specific co-expression modules, only the purple module detected in BA9 of the prefrontal cortex which is the most PD-specific module (Supplementary Figure S5) was significantly correlated with the light cyan module of AD *APOE* ε4 non-carriers with an extremely low *P*-value (**Figure 7A**, *P* = 9.522 × 10^-24^, Supplementary Table S26 for detailed overlapped genes). Two of the overlapping genes, *DUSP5* and *TBC1D8*, were hub genes of the light cyan module, and *DUSP5* was the top hub gene of the light cyan module (Supplementary Table S5). Furthermore, many annotations were the same between the two modules. The top diseases and functions and canonical pathways enriched in the light cyan module were also enriched in the purple module (**Figures 7B,C**). However, no BD-specific modules were found to be related to the three modules identified in AD (data not shown).

## Discussion

Unlike the analysis at the single-gene level, which can be influenced by co-expressed genes and thus give biased information, WGCNA, a systems biology approach which identifies co-expression gene modules, may better uncover the pathogenic and pathological mechanisms of complex diseases. In the present study, we identified two specific co-expression modules (violet and dark magenta) in AD *APOE* ε4 carriers and one specific module (light cyan) in AD *APOE* ε4 non-carriers. The most highly connected hub gene clusters (complete graph) were further identified in the violet and light cyan modules. Furthermore, the expression of these highly connected hub genes in the violet module was demonstrated to be correlated time-dependently under APOE ε4 treatment, whereas the expression of genes in the light cyan module was highly correlated time-dependently under APOE ε3 treatment. These experimental data verified the results of the WGCNA and further demonstrated the specificity and existence of the identified gene clusters.

The functional analysis of the identified modules in AD *APOE* ε4 carriers and non-carriers showed that the identified modules were enriched in distinct biological functions and signaling pathways, demonstrating that different biological processes could take part in disease progression with different *APOE* ε4 statuses, and thus they likely underlie distinctive clinical and pathological manifestations. Moreover, the expression patterns of conserved modules were the same in the AD *APOE* ε4 carriers and non-carriers. These results further highlight the significance of the three identified modules in the distinct characteristics between AD *APOE* ε4 carriers and non-carriers.

Our analysis demonstrated that the genes in both the violet and dark magenta modules of AD *APOE* ε4 carriers were strongly enriched in hereditary disorders, which was listed as the first among the diseases and functions of the IPA annotations. More importantly, a further analysis of enrichment with GWAS signals demonstrated that the expression patterns of these two modules had genetic bases in *APOE* ε4 carriers. Moreover, the significant SNPs of genes within both modules could not be replicated in *APOE* ε4 non-carriers. All these results demonstrated the genetic basis of *APOE* ε4 carriers for the development of AD.

The most highly connected hub gene cluster in the violet module was demonstrated to contain two phylogenetically conserved promoter motifs which could be targeted by some common TFs. Moreover, the mRNAs of these hub genes could be co-regulated by some common miRNAs. It is still unknown how the co-regulating feature in transcription is associated with the co-regulating feature in translation, however, the predicted conserved promoter motifs and common miRNAs suggest that some regulatory elements within these genes are phylogenetically conserved. This is consistent to the finding that the violet module has a genetic basis. Interestingly, three hub genes (*ENO3*, *ISOC1* and *GNB3*) in this cluster were enriched in the first annotation (hereditary disorder) and/or the second annotation (neurological disease) of diseases and functions. Moreover, they were three highly ranked hub genes of the violet module and some SNPs around them were *APOE* ε4 carrier-specific (Supplementary Tables S3 and S24). One SNP, rs5443 within *GNB3*, has been reported to correlate with increased APP expression (Bullido et al., 2004). In addition, this SNP is located within one of the ten most interesting genetic linkage regions related to AD (Guerreiro et al., 2012). Thus, we propose that these three genes could play important roles in AD *APOE* ε4 carriers with a genetic basis and also in a co-regulated manner.

Genes within the violet module of AD *APOE* ε4 carriers were mainly enriched in energy metabolism-associated signaling pathways. Energy metabolism and the expression of energy metabolism-associated genes are reported to be decreased in AD patients (Liang et al., 2008). However, the decrease in energy metabolism is even worse in *APOE* ε4 carriers who manifest it before AD development (Reiman et al., 2005; Wolf et al., 2013). Acetyl-CoA is a key intermediate in energy metabolism and also a key precursor for acetylcholine synthesis. It is almost exclusively synthesized in mitochondria from the pyruvate dehydrogenase complex (PDHC) reaction in the brain. It has been found that the activities of *PDHC* and choline acetyl transferase for acetylcholine synthesis are strongly suppressed in the brain cortexes of AD *APOE* ε4 carriers (Gibson et al., 2000; Bubber et al., 2005). Moreover, decreased acetyl-CoA for acetylcholine synthesis is responsible for decreased transmitter functions in cholinergic neurons of AD patients (Szutowicz et al., 2013). Furthermore, in mice expressing human APOE ε4, evoked hippocampal acetylcholine release is reduced (Dolejsi et al., 2016). Thus, our findings explain the pathological differences between AD *APOE* ε4 carriers and non-carriers.

Genes within the dark magenta module of AD *APOE* ε4 carriers were mainly enriched in GPCRs and their second messengers-associated signaling pathways. Actually, many GPCRs have been validated to take part in the pathological process of AD, such as muscarinic acetylcholine receptors (M receptors) and metabotropic glutamate receptors (Albasanz et al., 2005; Wess et al., 2007). M2 and M4 receptors seem to inhibit the soluble amino-terminal ectodomain of APP (sAPPα) release and potentially aggravate Aβ generation (Farber et al., 1995). Deletion of M1 receptors is responsible for increased amyloid pathology (Davis et al., 2010). However, some specific phenomena are observed in *APOE* ε4 carriers. The density of M2 receptor is specifically higher in some cerebral regions of healthy *APOE* ε4 carriers compared to non-carriers (Cohen et al., 2003). Facilitating brain noradrenergic and vasopressinergic activities improves cognitive function only in AD *APOE* ε4 carriers (Richard et al., 1997). Defects in GPCRs, especially M receptors, have been widely reported in AD; however, the related signaling pathways were specifically enriched in AD *APOE* ε4 carriers. Thus, we hypothesize that alterations in GPCRs could play critical pathogenic and pathological roles in *APOE* ε4 carriers.

The genes in the light cyan module identified in AD *APOE* ε4 non-carriers were enriched not only in neurological diseases but also in some other types of diseases, such as immunological and cardiovascular diseases with high rankings. Furthermore, many genes were enriched in all of these diseases. However, these phenomena were not observed in AD *APOE* ε4 carriers. The involvement of the immune system in the pathogenesis of AD has long been demonstrated (Akiyama, 1994). Cardiovascular diseases have been reported to share some common risk factors and common signaling pathways with AD (Martins et al., 2006). Moreover, this module was enriched in various signaling pathways. tRNA charging is part of protein synthesis, TR/RXR regulates gene expression, and TNFR1 is a receptor for TNFα which is a potent proinflammatory cytokine. ER stress, retinoid signaling and TNFR1 are reported to be associated with AD (Corcoran et al., 2004; de la Monte et al., 2012; Laske et al., 2013). ER stress could enhance the production of Aβ (Liu et al., 2014). ER stress is also involved in tauopathy (Sakagami et al., 2013). Activation of retinoid acid receptors and RXR can up-regulate α-secretase ADAM10 (Tippmann et al., 2009). RXR can form heterodimers with peroxisome proliferator-activated receptor γ (PPARγ), and their combined activation cooperatively enhances the microglial uptake of Aβ (Yamanaka et al., 2012). TR and RXR can up-regulate the mRNA of an AD-associated gene (Ishida et al., 2013). TNFR1 is up-regulated in AD brains and is required in Aβ-induced neuronal apoptosis and cognitive impairment (Li et al., 2004; Cheng et al., 2010; Lourenco et al., 2013). However, these signaling pathways are not limited to AD. They are also involved in many pathological processes of various diseases such as cardiovascular diseases, immunological diseases, PD, and inflammation (Yoshida, 2007; Van Hauwermeiren et al., 2011; Nizamutdinova et al., 2013). Thus, these data indicate that the development of AD in *APOE* ε4 non-carriers may not stem from unique pathogenic processes, but rather, AD shares some common pathways or develops concomitantly with other types of diseases. However, our findings do not preclude the probable effects of APOE ε4 on these signaling pathways, such as pathways of the immune systems, which have been previously demonstrated (Keene et al., 2011).

To further validate our results, we selected PD and BD, two brain diseases with some common clinical or pathological relationships with AD. Pathologically PD and AD are very similar, as both types of patients exhibit senile plaques and fibrillary tangles. Cognitive deficits in PD are attributed to the prefrontal cortex (Fuster, 2000), and PT and SN are responsible for extrapyramidal signs (Rinne et al., 1989). Our results demonstrated that the purple module detected in BA9 of the prefrontal cortex of PD patients was highly correlated with the light cyan module of AD *APOE* ε4 non-carriers, and such a correlation was not observed in PT and SN, suggesting the relationship of cognitive deficits between AD and PD. Thus, our data not only provide the evidence of the validity of the light cyan module but also demonstrate a relationship between PD and AD, especially the close relationship between PD and AD of *APOE* ε4 non-carriers. However, our data are not consistent with a previous study, which failed to detect any overlapping genes between AD and PD (Talwar et al., 2014). Because we demonstrated the difference between AD *APOE* ε4 carriers and non-carriers and because no risk genes were found in PD to play such a significant role as *APOE* ε4 in AD, the disparity could be attributed to the fact that the authors of the previous study did not stratify the AD samples according to *APOE* status. Although BD also shares some common characteristics with AD, especially at a later stage during disease progression, few studies have reported overlapping genes between AD and BD. In our study, disease-specific modules displayed no correlations between BD and AD. Thus, we propose that the clinical and pathological overlap between AD and BD could be due to downstream cascades from pathogenic factors of each disease. However, our study does not preclude the possible harmful effects of *APOE* ε4 on PD and BD which have been reported even though *APOE* ε4 is unlikely a strong risk factor for PD and BD (Soeira-de-Souza et al., 2010; Monsell et al., 2014).

The three modules identified did not show any evidence of correlation with aging (data not shown), the commonly acknowledged risk factor for AD. Infants carrying *APOE* ε4 have different temporal cortex structures compared to *APOE* ε4 non-carriers (Knickmeyer et al., 2014), and a similar difference was also observed in adults (Hua et al., 2008). Multiple approaches utilizing mice and humans have demonstrated that *APOE* ε4 can affect normal brain function even very early in life (DiBattista et al., 2016). Thus, the identified modules may exert roles in an age-independent manner which may take effect or promote disease progression at an early age.

In summary, the present study demonstrated that AD *APOE* ε4 carriers involve more genetic factors and that particular biological processes may exert pathogenic effects, whereas AD *APOE* ε4 non-carriers share more common pathways with other types of diseases, and AD may develop accompanying these diseases. Although some of the identified hub genes, signaling pathways, TFs and miRNAs are known to be AD-associated, our data further demonstrated that they are likely specifically associated with AD *APOE* ε4 carriers or non-carriers. Moreover, our study may help to uncover the function of poorly characterized hub gene(s), as WGCNA may reveal the functions of genes if they are in a module highly enriched in a particular biological process (Chen et al., 2013). Our study provides new insights into the pathogenic and pathological mechanisms other than those of *APOE4*, underlying the different characteristics of AD *APOE* ε4 carriers and non-carriers, which may open a new avenue for the further investigation of AD and might promote the differential treatment of AD based on *APOE* ε4 status.

## Author Contributions

SJ drafted the manuscript and contributed to the analytical approach and to the presentation and interpretation of the results. LT contributed to the experimental study and results analysis. NZ contributed to the experimental study. WY contributed to the analytical approach and discussion. YQ conceived of the study and contributed to the assembly and interpretation of the data, manuscript writing and the final approval of manuscript. H-ZC contributed to the critical review of manuscript, the supervision of the study and final approval of manuscript. All authors read and approved the final manuscript.

## Conflict of Interest Statement

The authors declare that the research was conducted in the absence of any commercial or financial relationships that could be construed as a potential conflict of interest.

## Footnotes

1 http://www.ncbi.nlm.nih.gov/geo/query/acc.cgi?acc=GSE15222

2 http://labs.genetics.ucla.edu/horvath/htdocs/CoexpressionNetwork/Rpackages/WGCNA/Tutorials/index.html

3 http://www.cytoscape.org

4 http://www.ingenuity.com

5 http://labs.med.miami.edu/myers/LFuN/data%20ajhg.html

6 http://www.ncbi.nlm.nih.gov/projects/gap/cgi-bin/study.cgi?study_id=phs000219.v1.p1

7 http://adni.loni.usc.edu/

8 http://pngu.mgh.harvard.edu/~purcell/plink/

9 http://www.ncbi.nlm.nih.gov/geo/query/acc.cgi?acc=GSE20295

10 http://www.ncbi.nlm.nih.gov/geo/query/acc.cgi?acc=GSE12654
