# Supplementary material for: A Systems View of the Differences between APOE ε4 Carriers and Non-carriers in Alzheimer’s Disease
Source: Front Aging Neurosci. 2016 Jul 12;8:171. doi: 10.3389/fnagi.2016.00171 (PMC4941795; doi:10.3389/fnagi.2016.00171)
Supplement: Supplementary file 1 [file Data_Sheet_1.PDF]

Supplementary Material

A systems view of the differences between APOE ε4 carriers and non-carriers in Alzheimer's disease

Shan Jiang, Ling Tang, Na Zhao, Wanling Yang, Zhen-yu Pan, Yu Qiu, Hong-zhuan Chen

*correspondence: Yu Qiu, yu_qiu@hotmail.com; Hong-Zhuan Chen hongzhuan_chen@hotmail.com

Supplementary Figures

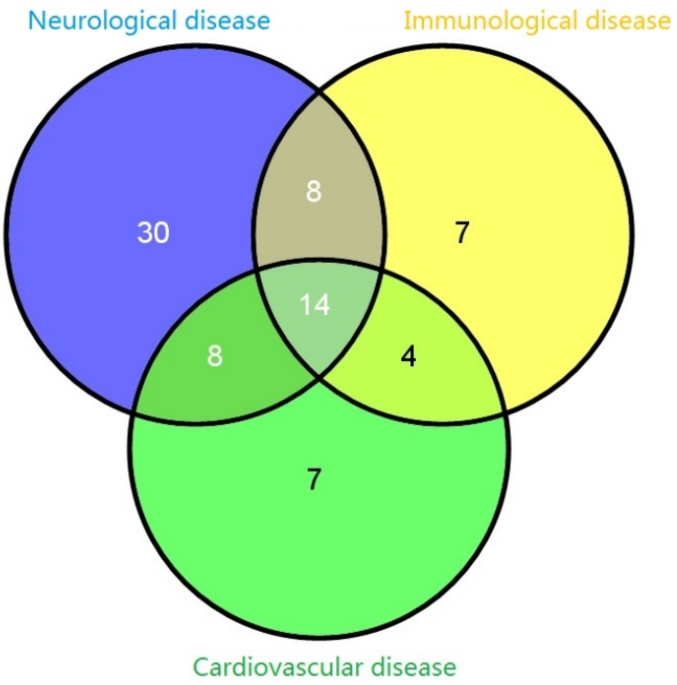

Supplementary Figure S1. Venn diagram of overlapped enriched gene numbers among neurological, immunological and cardiovascular diseases in AD *APOE* ε4 non-carriers.

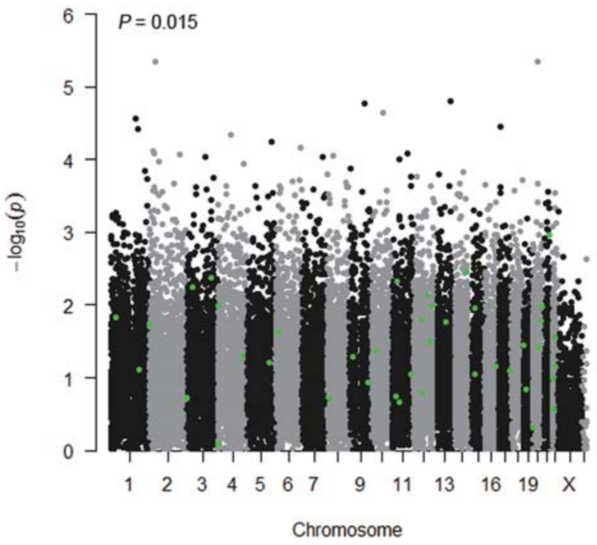

Supplementary Figure S2. Manhattan plot of enrichment analyses of genetic association signals from GenADA data for the dark magenta module of AD *APOE* ε4 carriers. The green dots represent the genes within dark magenta module.

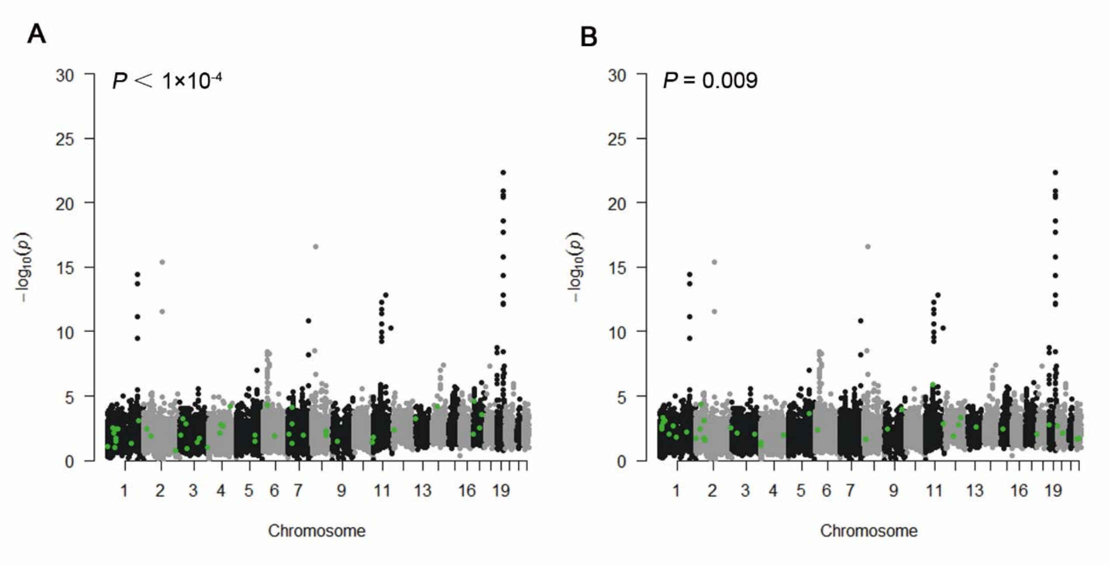

Supplementary Figure S3. Manhattan plots of enrichment analyses of genetic association signals from IGAP data for the violet and dark magenta modules of AD *APOE* ε4 carriers. The green dots represent the genes within violet (a) and darkmagenta (b) modules.

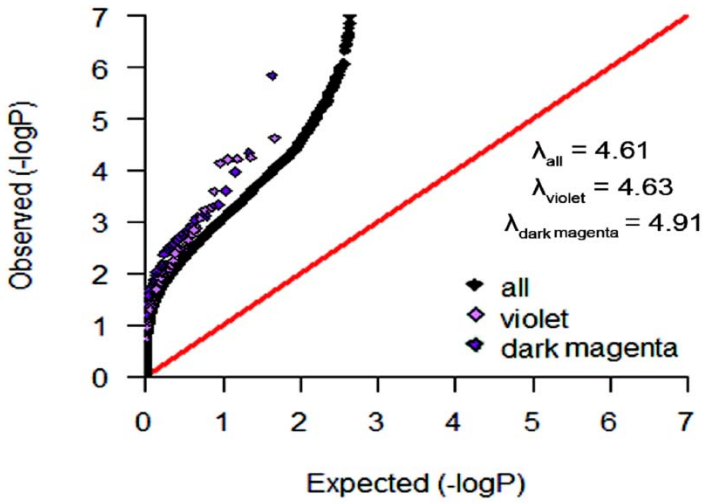

Supplementary Figure S4. comparative QQ plots of genetic association signals for the violet and dark magenta modules of AD *APOE* ε4 carriers. The black dots represent all genes, violet dots represent the genes within violet module and dark magenta represents the genes within dark magenta module. Values of genomic dispersion factor λ were given in the middle-right of comparative QQ plot.

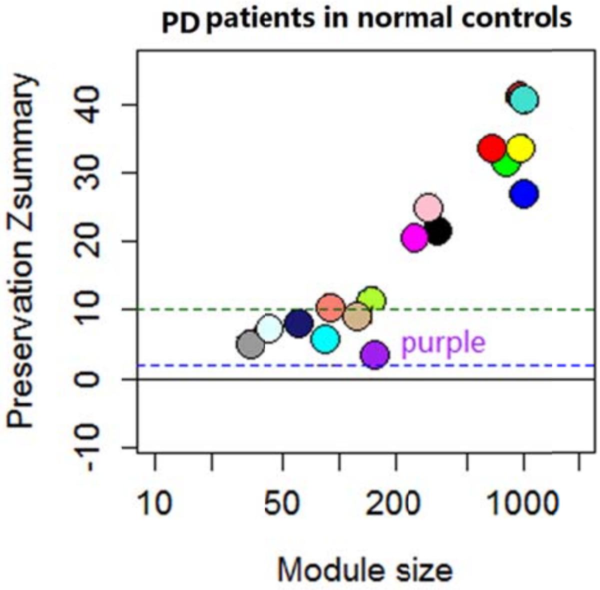

Supplementary Figure S5. Preservation statistic of the modules of PD patients in normal controls detected in BA9 of prefrontal cortex. The thresholds $Z_{summary}$ = 2 and $Z_{summary}$ = 10 were indicated by dashed blue and green lines respectively. $Z_{summary}$ less than 2 implies no evidence of module preservation, $Z_{summary}$ between 2 and 10 implies modest evidence of module preservation and $Z_{summary}$ more than 10 implies strong evidence of module preservation.

Supplementary Tables

Supplementary Table S1. Demographic characteristics of the participants for WGCNA

Characteristic	*APOE* ε 4 non-carriers Controls (n = 148)	*APOE* ε 4 non-carriers AD patients (n = 54)	*APOE* ε4 carriers Controls (n = 40)	*APOE* ε4 carriers AD patients (n = 121)	statistic	*P* value
Age, year, median ± QR	82.5 ± 14	84 ± 13	80.5 ± 13.75	83 ± 8	χ^2 = 3.500[a]	0.321[a]
Female, %	46.6	41.0	55.6	46.7	χ^2 = 2.138[b]	0.544[b]
PMI, hour, median ± QR	6.678 ± 8.5	6.615 ± 10.025	4.69 ± 9.31	4 ± 6.83	χ^2 = 0.668[a]	0.881[a]
Percent of samples from each institute, %, mean ± SEM	0.056 ± 0.010	0.083 ± 0.059	0.077 ± 0.044	0.046 ± 0.012	F = 0.957[c]	0.420[c]
Hybridization date, day, median ± QR	4 ± 5	5 ± 5	4 ± 4.75	4 ± 6	χ^2 = 1.035[a]	0.793[a]

[a]Brown-Mood nonparametric chi-square test.
[b]chi-square test.
[c]ANOVA.

Supplementary Table S2. Summary of *Zsummary* statistics of the sub-dataset-specific co-expression modules in either AD *APOE* ε4 carriers or AD *APOE* ε4 non-carriers

module	Module preservation	*Zsummary* statistics[a]
Violet module of AD *APOE* ε4 carriers	AD *APOE* ε4 carriers in AD *APOE* ε4 non-carriers	0.57
	AD *APOE* ε4 carriers in control *APOE* ε4 carriers	0.67
Darkmagenta module of AD *APOE* ε4 carriers	AD *APOE* ε4 carriers in AD *APOE* ε4 non-carriers	1.10
	AD *APOE* ε4 carriers in control *APOE* ε4 carriers	0.25
Lightcyan module of AD *APOE* ε4 non-carriers	AD *APOE* ε4 non-carriers in AD *APOE* ε4 carriers	-0.19
	AD *APOE* ε4 non-carriers in control *APOE* ε4 non-carriers	1.8

[a] $Z_{summary}$ less than 2 implies no evidence of module preservation, which indicates strong evidence of module specificity. $Z_{summary}$ between 2 and 10 implies modest evidence of module preservation and $Z_{summary}$ more than 10 implies strong evidence of module preservation, which indicates modest or no evidence of module specificity.

Supplementary Table S6. Pearson correlation coefficient matrix for the top six hub genes comprising the complete graph within the violet module of AD *APOE* ε4 carriers

	ENO3	*GNB3*	*XPO4*	*GDF10*	*ISOC1*	*ACLY*
ENO3	1	0.9680712	0.9234593	0.9188684	0.8808758	0.7849963
GNB3	0.9680712	1	0.9453785	0.9053457	0.8697317	0.8053816
XPO4	0.9234593	0.9453785	1	0.9072076	0.8920728	0.8396081
GDF10	0.9188684	0.9053457	0.9072076	1	0.9075149	0.8438105
ISOC1	0.8808758	0.8697317	0.8920728	0.9075149	1	0.899367
ACLY	0.7849963	0.8053816	0.8396081	0.8438105	0.899367	1

Supplementary Table S7. Pearson correlation coefficient matrix for the top sixteen hub genes comprising the complete graph within the light cyan module of AD *APOE* ε4 non-carriers

	PLIN2	*CSNK1D*	*ATF5*	*CHST12*	*ST8SIA5*	*BDNF*	*ERRFI1*	*MIR7-3HG*	*TBC1D8*	*HILPDA*	*SYT12*	*RIN1*	*DNAJB5*	*ZNF331*	*TNFRSF18*	*DUSP5*
PLIN2	1	0.8458982	0.9031487	0.7976189	0.6951779	0.7229838	0.8078227	0.8248706	0.8357988	0.9308506	0.7935344	0.8655363	0.8493243	0.8572307	0.8758856	0.8705149
CSNK1D	0.8458982	1	0.8180747	0.9004921	0.823491	0.8658587	0.8868207	0.8187085	0.8898859	0.8870648	0.8699575	0.8412129	0.873281	0.8942266	0.9039065	0.9091074
ATF5	0.9031487	0.8180747	1	0.8221893	0.767869	0.7852795	0.8173841	0.8654846	0.8140467	0.9104862	0.8702278	0.9233979	0.8844879	0.9005579	0.9092319	0.9050756
CHST12	0.7976189	0.9004921	0.8221893	1	0.873779	0.8681603	0.8900479	0.7817599	0.8702654	0.8613096	0.9257443	0.8946444	0.9124128	0.8888512	0.898059	0.9138648
ST8SIA5	0.6951779	0.823491	0.767869	0.873779	1	0.9215115	0.8658693	0.8162876	0.8793197	0.7927789	0.9434796	0.8615596	0.9013688	0.8942697	0.8708309	0.9038893
BDNF	0.7229838	0.8658587	0.7852795	0.8681603	0.9215115	1	0.9366784	0.7456294	0.8823521	0.7662783	0.8511305	0.8008304	0.8765985	0.8932459	0.841101	0.8678191
ERRFI1	0.8078227	0.8868207	0.8173841	0.8900479	0.8658693	0.9366784	1	0.8235972	0.9306402	0.8710267	0.8730529	0.8610546	0.8915345	0.9412769	0.9064311	0.9319983
MIR7-3HG	0.8248706	0.8187085	0.8654846	0.7817599	0.8162876	0.7456294	0.8235972	1	0.8601428	0.9330097	0.9082743	0.9302664	0.8857001	0.9135508	0.9294902	0.9429743
TBC1D8	0.8357988	0.8898859	0.8140467	0.8702654	0.8793197	0.8823521	0.9306402	0.8601428	1	0.8836124	0.872309	0.8492074	0.8966804	0.922081	0.9001356	0.9295263
HILPDA	0.9308506	0.8870648	0.9104862	0.8613096	0.7927789	0.7662783	0.8710267	0.9330097	0.8836124	1	0.8953835	0.9401552	0.8946504	0.9402266	0.9440517	0.9543812
SYT12	0.7935344	0.8699575	0.8702278	0.9257443	0.9434796	0.8511305	0.8730529	0.9082743	0.872309	0.8953835	1	0.9503286	0.9552074	0.933984	0.9336488	0.9627272
RIN1	0.8655363	0.8412129	0.9233979	0.8946444	0.8615596	0.8008304	0.8610546	0.9302664	0.8492074	0.9401552	0.9503286	1	0.9441452	0.949565	0.9649019	0.9560673
DNAJB5	0.8493243	0.873281	0.8844879	0.9124128	0.9013688	0.8765985	0.8915345	0.8857001	0.8966804	0.8946504	0.9552074	0.9441452	1	0.9453993	0.9196395	0.9622348
ZNF331	0.8572307	0.8942266	0.9005579	0.8888512	0.8942697	0.8932459	0.9412769	0.9135508	0.922081	0.9402266	0.933984	0.949565	0.9453993	1	0.9585241	0.9853452
TNFRSF18	0.8758856	0.9039065	0.9092319	0.898059	0.8708309	0.841101	0.9064311	0.9294902	0.9001356	0.9440517	0.9336488	0.9649019	0.9196395	0.9585241	1	0.9532068
DUSP5	0.8705149	0.9091074	0.9050756	0.9138648	0.9038893	0.8678191	0.9319983	0.9429743	0.9295263	0.9543812	0.9627272	0.9560673	0.9622348	0.9853452	0.9532068	1

Supplementary Table S8. Summarized counts of random samplings which replicated the three sub-dataset-specific modules detected in total samples*

Module	50% random sampling	75% random sampling
Violet in AD *APOE* ε4 carriers	2	3
Darkmagenta in AD *APOE* ε4 carriers	3	5
Lightcyan in AD *APOE* ε4 non-carriers	2	3

*Each level of random samplings was performed for five times, and numbers in the cells of cross table indicate the replicated counts of the corresponding modules in the corresponding random sampling level.

Supplementary Table S9. Pearson correlation coefficient matrix for the time-dependent mRNA expression of the six genes within the complete graph of the violet module of AD *APOE* ε4 carriers in primary cultured neurons treated with recombinant human APOE ε4

	EN03	GNB3	XPO4	GDF10	ISOC1	ACLY
EN03	1	0.7269543	0.8222588	0.9872821	0.7962731	0.7607974
GNB3	0.7269543	1	0.9885469	0.8268772	0.994256	0.9987168
XPO4	0.8222588	0.9885469	1	0.9022781	0.9990207	0.9949212
GDF10	0.9872821	0.8268772	0.9022781	1	0.8823181	0.854297
ISOC1	0.7962731	0.994256	0.9990207	0.8823181	1	0.9984004
ACLY	0.7607974	0.9987168	0.9949212	0.854297	0.9984004	1

Supplementary Table S10. Pearson correlation coefficient matrix for the time-dependent mRNA expression of the six genes within the complete graph of the violet module of AD *APOE* ε4 carriers in primary cultured neurons treated with recombinant human APOE ε3

	EN03	GNB3	XPO4	GDF10	ISOC1	ACLY
EN03	1	0.519192	-0.83736	0.9190891	0.8602825	-0.65116
GNB3	0.519192	1	0.0324488	0.140406	0.8823714	0.3105577
XPO4	-0.83736	0.0324488	1	-0.985017	-0.441674	0.9601312
GDF10	0.9190891	0.140406	-0.985017	1	0.5897826	-0.897534
ISOC1	0.8602825	0.8823714	-0.441674	0.5897826	1	-0.17326
ACLY	-0.65116	0.3105577	0.9601312	-0.897534	-0.17326	1

Supplementary Table S11. Pearson correlation coefficient matrix for the time-dependent mRNA expression of the six genes within the complete graph of the violet module of AD *APOE* ε4 carriers in primary cultured neurons without APOE treatment

	EN03	GNB3	XPO4	GDF10	ISOC1	ACLY
EN03	1	-0.225132	-0.653624	0.9739149	0.2105526	0.3655399
GNB3	-0.225132	1	0.8845422	0.0018286	0.9050841	0.8246057
XPO4	-0.653624	0.8845422	1	-0.464842	0.602231	0.4655183
GDF10	0.9739149	0.0018286	-0.464842	1	0.4268869	0.5672147
ISOC1	0.2105526	0.9050841	0.602231	0.4268869	1	0.986895
ACLY	0.3655399	0.8246057	0.4655183	0.5672147	0.986895	1

Supplementary Table S12. Pearson correlation coefficient matrix for the time-dependent mRNA expression of the twelve genes within the complete graph of the light cyan module of AD *APOE* ε4 non-carriers in primary cultured neurons treated with recombinant human APOE ε3

	DUSP5	TNFRSF18	DNAJB5	SYT12	HILPDA	TBC1D8	BDNF	ST8SIA5	CHST12	ATF5	CSNK1D	PLIN2
DUSP5	1	-0.792171	-0.698477	-0.567693	-0.926509	-0.999994	0.9802474	0.9943784	0.7947686	0.9751389	0.9531606	-0.564939
TNFRSF18	-0.792171	1	0.9900633	0.9521329	0.9635921	0.7901154	-0.897225	-0.723096	-0.999991	-0.637238	-0.93966	0.9511064
DNAJB5	-0.698477	0.9900633	1	0.9856579	0.9164182	0.6960688	-0.826215	-0.618776	-0.989454	-0.522533	-0.882215	0.9850886
SYT12	-0.567693	0.9521329	0.9856579	1	0.8357349	0.5649231	-0.719296	-0.477333	-0.950819	-0.371154	-0.790103	0.9999944
HILPDA	-0.926509	0.9635921	0.9164182	0.8357349	1	0.9252396	-0.982626	-0.881459	-0.964725	-0.820095	-0.996921	0.8338959
TBC1D8	-0.999994	0.7901154	0.6960688	0.5649231	0.9252396	1	-0.979577	-0.994729	-0.792725	-0.975878	-0.952139	0.5621636
BDNF	0.9802474	-0.897225	-0.826215	-0.719296	-0.982626	-0.979577	1	0.9537955	0.8991021	0.9120516	0.9941532	-0.716971
ST8SIA5	0.9943784	-0.723096	-0.618776	-0.477333	-0.881459	-0.994729	0.9537955	1	0.726038	0.9931206	0.915776	-0.474395
CHST12	0.7947686	-0.999991	-0.989454	-0.950819	-0.964725	-0.792725	0.8991021	0.726038	1	0.6405215	0.9411117	-0.949779
ATF5	0.9751389	-0.637238	-0.522533	-0.371154	-0.820095	-0.975878	0.9120516	0.9931206	0.6405215	1	0.8624396	-0.36805
CSNK1D	0.9531606	-0.93966	-0.882215	-0.790103	-0.996921	-0.952139	0.9941532	0.915776	0.9411117	0.8624396	1	-0.788051
PLIN2	-0.564939	0.9511064	0.9850886	0.9999944	0.8338959	0.5621636	-0.716971	-0.474395	-0.949779	-0.36805	-0.788051	1

Supplementary Table S13. Pearson correlation coefficient matrix for the time-dependent mRNA expression of the twelve genes within the complete graph of the light cyan module of AD *APOE* ε4 non-carriers in primary cultured neurons treated with recombinant human APOE ε4

	DUSP5	TNFRSF18	DNAJB5	SYT12	HILPDA	TBC1D8	BDNF	ST8SIA5	CHST12	ATF5	CSNK1D	PLIN2
DUSP5	1	0.7924825	0.9144965	0.7334978	0.9961744	0.7178881	-0.565	-0.980702	0.4741499	0.279128	0.4115811	0.3520026
TNFRSF18	0.7924825	1	0.4779629	0.9958246	0.7361536	0.9934971	-0.950971	-0.89643	-0.161223	-0.36445	-0.229671	-0.291905
DNAJB5	0.9144965	0.4779629	1	0.3957821	0.9463544	0.3748448	-0.182864	-0.817746	0.7898306	0.6437743	0.7451254	0.7006045
SYT12	0.7334978	0.9958246	0.3957821	1	0.6712952	0.9997426	-0.975234	-0.85223	-0.250643	-0.447937	-0.317559	-0.377998
HILPDA	0.9961744	0.7361536	0.9463544	0.6712952	1	0.6543062	-0.490736	-0.959865	0.5492758	0.3619743	0.4896491	0.4324506
TBC1D8	0.7178881	0.9934971	0.3748448	0.9997426	0.6543062	1	-0.980001	-0.840141	-0.272542	-0.468106	-0.338991	-0.398905
BDNF	-0.565	-0.950971	-0.182864	-0.975234	-0.490736	-0.980001	1	0.7154106	0.4585519	0.6345896	0.5194226	0.5734029
ST8SIA5	-0.980702	-0.89643	-0.817746	-0.85223	-0.959865	-0.840141	0.7154106	1	-0.292863	-0.086001	-0.225455	-0.162211
CHST12	0.4741499	-0.161223	0.7898306	-0.250643	0.5492758	-0.272542	0.4585519	-0.292863	1	0.9777984	0.9975643	0.9909969
ATF5	0.279128	-0.36445	0.6437743	-0.447937	0.3619743	-0.468106	0.6345896	-0.086001	0.9777984	1	0.9900334	0.9970505
CSNK1D	0.4115811	-0.229671	0.7451254	-0.317559	0.4896491	-0.338991	0.5194226	-0.225455	0.9975643	0.9900334	1	0.997922
PLIN2	0.3520026	-0.291905	0.7006045	-0.377998	0.4324506	-0.398905	0.5734029	-0.162211	0.9909969	0.9970505	0.997922	1

Supplementary Table S14. Pearson correlation coefficient matrix for the time-dependent mRNA expression of the twelve genes within the complete graph of the light cyan module of AD *APOE* ε4 non-carriers in primary cultured neurons without APOE treatment

	DUSP5	TNFRSF18	DNAJB5	SYT12	HILPDA	TBC1D8	BDNF	ST8SIA5	CHST12	ATF5	CSNK1D	PLIN2
DUSP5	1	-0.024735	0.9098273	0.8174559	0.5760093	0.5489141	-0.985988	0.9999982	-0.071179	-0.976208	0.4625302	-0.440137
TNFRSF18	-0.024735	1	0.3923553	0.5555948	0.8029452	0.8220454	0.1911558	-0.022817	0.998919	0.2409147	0.8748914	0.908543
DNAJB5	0.9098273	0.3923553	1	0.9827726	0.8632974	0.846296	-0.827851	0.9106219	0.3491742	-0.798198	0.7887517	-0.027818
SYT12	0.8174559	0.5555948	0.9827726	1	0.9417023	0.9301719	-0.709916	0.8185597	0.5163447	-0.673113	0.8887739	0.157408
HILPDA	0.5760093	0.8029452	0.8632974	0.9417023	1	0.999463	-0.431574	0.577577	0.7743701	-0.385056	0.9911697	0.4804846
TBC1D8	0.5489141	0.8220454	0.846296	0.9301719	0.999463	1	-0.401783	0.5505171	0.7946876	-0.354607	0.9949824	0.5089642
BDNF	-0.985988	0.1911558	-0.827851	-0.709916	-0.431574	-0.401783	1	-0.985666	0.2365763	0.9987014	-0.308148	0.5837603
ST8SIA5	0.9999982	-0.022817	0.9106219	0.8185597	0.577577	0.5505171	-0.985666	1	-0.069265	-0.975791	0.4642308	-0.438413
CHST12	-0.071179	0.998919	0.3491742	0.5163447	0.7743701	0.7946876	0.2365763	-0.069265	1	0.2857695	0.8514324	0.9269817
ATF5	-0.976208	0.2409147	-0.798198	-0.673113	-0.385056	-0.354607	0.9987014	-0.975791	0.2857695	1	-0.25928	0.6243671
CSNK1D	0.4625302	0.8748914	0.7887517	0.8887739	0.9911697	0.9949824	-0.308148	0.4642308	0.8514324	-0.25928	1	0.5925322
PLIN2	-0.440137	0.908543	-0.027818	0.157408	0.4804846	0.5089642	0.5837603	-0.438413	0.9269817	0.6243671	0.5925322	1

Supplementary Table S24. Significant SNPs within 20 kb gene boundaries of the violet module in *APOE* ε4 carriers

SNP rs number	20 kb boundary of gene belonged to	*P*-value in carriers	*P*-value in non-carriers	GWAS dataset belonged to
rs181405	*BCL2L13*	0.01277	0.8607	ADNI
rs3758653	*DRD4*	0.008013	0.03123	ADNI
rs238238	*ENO3*	0.04366	0.3572	ADNI
rs17682361	*ESRRG*	0.002383	0.05132	ADNI
rs12089054	*ESRRG*	0.02272	0.1007	ADNI
rs12077706	*ESRRG*	0.01387	0.1037	ADNI
rs10495034	*ESRRG*	0.007955	0.1066	ADNI
rs17695327	*ESRRG*	0.03967	0.1116	ADNI
rs871557	*ESRRG*	0.0412	0.2056	ADNI
rs830322	*ESRRG*	0.02464	0.2584	ADNI
rs4846812	*ESRRG*	0.02337	0.2962	ADNI
rs1354221	*ESRRG*	0.006755	0.376	ADNI
rs1561186	*ESRRG*	0.009361	0.3958	ADNI
rs1542003	*ESRRG*	0.02767	0.4122	ADNI
rs1166332	*ESRRG*	0.001553	0.4786	ADNI
rs830307	*ESRRG*	0.02986	0.4888	ADNI
rs11117697	*ESRRG*	0.02602	0.6501	ADNI
rs6696627	*ESRRG*	0.005395	0.6906	ADNI
rs12119765	*ESRRG*	0.03394	0.7449	ADNI
rs10492953	*ESRRG*	0.02683	0.8234	ADNI
rs2789725	*ESRRG*	0.01595	0.8685	ADNI
rs6673566	*ESRRG*	0.03867	0.9257	ADNI
rs830308	*ESRRG*	0.001264	0.9611	ADNI
rs10516809	*HERC5*	0.04907	0.1561	ADNI
rs7624803	*HSPBAP1*	0.04267	0.3276	ADNI
rs9812646	*HSPBAP1*	0.02465	0.4359	ADNI
rs3732832	*HSPBAP1*	0.008479	0.4601	ADNI
rs2137210	*HSPBAP1*	0.03983	0.6961	ADNI
rs4836437	*ISOC1*	0.01105	0.2268	ADNI
rs3798111	*ISOC1*	0.01441	0.2352	ADNI
rs10849196	*KCNA5*	0.02678	0.3301	ADNI
rs7017753	*LAPTM4B*	0.03567	0.1237	ADNI
rs2449545	*LAPTM4B*	0.02619	0.7735	ADNI
rs2512033	*MATN2*	0.02961	0.04289	ADNI
rs2444876	*MATN2*	0.02443	0.04962	ADNI
rs2444882	*MATN2*	0.03127	0.06433	ADNI
rs2085144	*MATN2*	0.03813	0.2822	ADNI
rs2061054	*MATN2*	0.00983	0.4259	ADNI
rs964141	*MATN2*	0.01477	0.5941	ADNI
rs2061052	*MATN2*	0.0394	0.8195	ADNI

rs2255317	*MATN2*	0.0394	0.836	ADNI
rs17833955	*MSI2*	0.0394	0.04307	ADNI
rs277068	*MSI2*	0.04902	0.09807	ADNI
rs1477065	*MSI2*	0.02	0.1717	ADNI
rs8067335	*MSI2*	0.01859	0.2313	ADNI
rs930521	*MSI2*	0.03965	0.3237	ADNI
rs888117	*MSI2*	0.02304	0.5562	ADNI
rs1561945	*MSI2*	0.04271	0.6047	ADNI
rs6503802	*MSI2*	0.02654	0.8758	ADNI
rs2276847	*SELK*	0.000238	0.3023	ADNI
rs3916033	*SERPINF1*	0.0451	0.6127	ADNI
rs688630	*SGIP1*	0.04769	0.07064	ADNI
rs7515848	*SGIP1*	0.01894	0.2822	ADNI
rs11208952	*SGIP1*	0.02884	0.4746	ADNI
rs9659684	*SGIP1*	0.03029	0.8847	ADNI
rs17418864	*TMCO1*	0.03861	0.2844	ADNI
rs1034472	*BCL2L13*	0.001883	0.3613	GenADA
rs9617611	*BCL2L13*	0.03032	0.7364	GenADA
rs11244667	*DHX32*	0.01331	0.02072	GenADA
rs11244672	*DHX32*	0.005405	0.1731	GenADA
rs10144981	*ERO1L*	0.00679	0.7209	GenADA
rs4532915	*ESRRG*	0.004021	0.1275	GenADA
rs6698423	*ESRRG*	0.0342	0.1821	GenADA
rs6681237	*ESRRG*	0.008975	0.6415	GenADA
rs7538474	*ESRRG*	0.007031	0.7608	GenADA
rs11204215	*GDF10*	0.01611	0.1358	GenADA
rs878263	*GDF10*	0.02743	0.1928	GenADA
rs9534034	*GTF2F2*	0.03267	0.1587	GenADA
rs17066496	*GTF2F2*	0.02127	0.7626	GenADA
rs9812880	*HRASLS*	0.02201	0.08515	GenADA
rs10804951	*HRASLS*	0.03375	0.4047	GenADA
rs10511415	*HSPBAP1*	0.04473	0.4746	GenADA
rs10849196	*KCNA5*	0.03989	0.9353	GenADA
rs6416319	*KCNA5*	0.02718	0.3659	GenADA
rs11063476	*KCNA5*	0.03787	0.8519	GenADA
rs11063477	*KCNA5*	0.01767	0.8931	GenADA
rs4735498	*LAPTM4B*	0.0472	0.1182	GenADA
rs4735508	*MATN2*	0.02836	0.7523	GenADA
rs11997426	*MATN2*	0.01726	0.2901	GenADA
rs4583310	*MSI2*	0.01114	0.239	GenADA
rs7208507	*MSI2*	0.04802	0.7623	GenADA
rs7208395	*MSI2*	0.02768	0.8133	GenADA
rs2333025	*MSI2*	0.0312	0.5673	GenADA
rs276695	*MYO6*	0.01374	0.5532	GenADA

rs276699	*MYO6*	0.003676	1	GenADA
rs12508946	*SCARB2*	0.0054	0.8514	GenADA
rs1325266	*SGIP1*	0.003461	0.136	GenADA
rs1325267	*SGIP1*	0.01083	0.1388	GenADA
rs1325268	*SGIP1*	0.01694	0.1963	GenADA
rs920987	*SGIP1*	0.0224	0.3916	GenADA
rs17434186	*SGIP1*	0.01989	0.7931	GenADA
rs17418864	*TMCO1*	0.03086	0.3709	GenADA
rs41528748	*TMEM59*	0.04624	0.4948	GenADA
rs11743931	*ZNF608*	0.0331	0.08089	GenADA
rs7727757	*ZNF608*	0.01372	0.2177	GenADA
rs4240378	*ZNF608*	0.04601	N/A[a]	GenADA
rs3744779	*ACLY*	0.01398	0.8466	Myer
rs5747296	*BCL2L13*	0.02223	0.6891	Myer
rs5747338	*BCL2L13*	0.007041	0.8816	Myer
rs7793849	*CBX3*	0.0004444	0.525	Myer
rs11604855	*DRD4*	0.01273	0.4704	Myer
rs2243102	*ENO3*	0.03912	0.009186	Myer
rs7548662	*ESRRG*	0.04644	0.8758	Myer
rs6686952	*ESRRG*	0.01044	0.2618	Myer
rs4846400	*ESRRG*	0.01548	0.07217	Myer
rs1436899	*ESRRG*	0.006296	0.4481	Myer
rs743508	*GDF10*	0.03337	0.3917	Myer
rs1902713	*GDF10*	0.03429	0.7114	Myer
rs17295858	*GTF2F2*	0.01963	0.1372	Myer
rs4483759	*GTF2F2*	0.04534	0.3128	Myer
rs4766333	*KCNA5*	0.03208	0.6023	Myer
rs7965690	*KCNA5*	0.03364	0.7124	Myer
rs963999	*KIAA0556*	0.03066	0.9095	Myer
rs12447401	*KIAA0556*	0.03798	0.3249	Myer
rs1136595	*LAPTM4B*	0.04303	0.4112	Myer
rs16896340	*LAPTM4B*	0.0005925	0.7225	Myer
rs16896476	*MATN2*	0.02238	0.68	Myer
rs2513816	*MATN2*	0.02674	0.3899	Myer
rs1136595	*MATN2*	0.04303	0.4112	Myer
rs6995084	*MATN2*	0.002027	0.4058	Myer
rs11868089	*MSI2*	0.008959	0.1802	Myer
rs6503808	*MSI2*	0.04813	0.7501	Myer
rs792387	*MSI2*	0.03477	0.2335	Myer
rs12196105	*MYO6*	0.03871	0.9631	Myer
rs2295935	*MYO6*	0.02855	N/A[a]	Myer
rs12210386	*MYO6*	0.03908	0.07036	Myer
rs2208798	*MYO6*	0.04526	0.5568	Myer
rs10187066	*PLCD4*	0.02124	0.5979	Myer

rs4655625	*SGIP1*	0.03844	0.7295	Myer
rs10789215	*SGIP1*	0.02912	0.4218	Myer
rs17129365	*SGIP1*	0.04297	0.2727	Myer
rs6689451	*SGIP1*	0.00404	0.4042	Myer
rs7330667	*XPO4*	0.03448	0.465	Myer

[a]N/A indicates not applicable because the SNPs did not pass quality control.

Supplementary Table S25. Identified miRNAs which can bind to mRNA 3'UTR of the five hub genes within the complete graph of the violet module of AD *APOE* ε4 carriers*

	ISOC1	*ENO3*	*GDF10*	*XPO4*	*ACLY*
hsa-miR-194	miRanda/Targetscan	miRanda/miRWalk	miRanda/miRWalk	miRanda/miRWalk	miRanda/Targetscan
hsa-miR-199a-5p	miRanda	miRanda/miRWalk/Targetscan	miRanda/miRWalk	miRanda/miRWalk/Targetscan	miRanda/miRWalk
hsa-miR-199b-5p	miRanda	miRanda/miRWalk/Targetscan	miRanda/miRWalk	miRanda/miRWalk/Targetscan	miRanda/miRWalk
hsa-miR-30a	miRanda/miRWalk	miRanda	miRanda/miRWalk	miRanda/miRWalk/Targetscan	miRanda/miRWalk
hsa-miR-30d	miRanda/miRWalk	miRanda	miRanda/miRWalk	miRanda/miRWalk/Targetscan	miRanda/miRWalk
hsa-miR-30e	miRanda/miRWalk	miRanda	miRanda/miRWalk	miRanda/miRWalk/Targetscan	miRanda/miRWalk

* miRNA prediction programs in the cell of the cross table indicates that the miRNA in the row can bind to 3'UTR of mRNA of the gene in the column.

Supplementary Table S26. Genes of the purple module in BA9 of the prefrontal cortex of PD patients

Genes in purple module	overlapped with lightcyan module
VCL	yes
SCML1	yes
N4BP2L2	yes
CD44	yes
RGS16	yes
PDPN	yes
GADD45B	yes
BAG3	yes
EGR1	yes
FOS	yes
ZFP36	yes
NFIL3	yes
LDLR	yes
FOSB	yes
FOSL2	yes
TBC1D8	yes
DDIT3	yes
XBP1	yes
SOD2	yes
PDE4B	yes
PTP4A1	yes
CDKN1A	yes
DUSP5	yes
SLC47A1	no
BTG3	no
GADD45A	no
ZNF410	no
HSPA1A	no
USPL1	no
IFI16	no
SLC2A3	no
TRIM23	no
SHC1	no
ANGPTL4	no
CORO1C	no
FKBP4	no
HSD17B7	no
DUSP6	no
FAM171A1	no
PKIG	no
PIGA	no

SEC31B	no
RBM3	no
MGP	no
SP110	no
LIMK2	no
NIP7	no
HSPA4	no
PRKD2	no
CLIC1	no
TRAF3IP2	no
PNP	no
CD55	no
NINJ1	no
COLEC12	no
SAT1	no
VAMP5	no
IL4R	no
PLEKHO2	no
CEBPD	no
YAP1	no
GPR56	no
CHST3	no
HPS5	no
MCL1	no
SMA4	no
COL4A1	no
SLC31A2	no
BRD4	no
APOBEC3A	no
RANBP3	no
ARHGEF12	no
PIAS1	no
TINF2	no
PDS5A	no
ZNF44	no
VPS33A	no
MAFF	no
CEBPB	no
MLLT3	no
GALNT2	no
HBEGF	no
CYP1B1	no
JUN	no
HSPB2	no

DNAJB1	no
RNMT	no
CD93	no
MPZL2	no
PDE6B	no
ASAP3	no
RELA	no
HSPB1	no
LAPTM5	no
RASSF1	no
ASXL1	no
PTPN1	no
PLCXD1	no
MPZL1	no
MAPK1IP1L	no
MYOF	no
ANXA2	no
TIPARP	no
BNIP2	no
PLOD2	no
TTC28	no
PTBP1	no
BRAP	no
HNRNPU	no
SNRPB	no
P4HA1	no
LSM2	no
NUDCD3	no
NEUROD2	no
BAZ1A	no
SHMT2	no
FEM1C	no
AKAP12	no
CXCL14	no
C10orf10	no
PALM2-AKAP2	no
LRRFIP1	no
FBXL14	no
HSPD1	no
RCAN1	no
SLC39A7	no
RBMS1	no
HPS6	no
SPRY2	no

LTBP1	no

Supplementary Acknowledgements

We thank investigators and managers within ADNI for offering ADNI GWAS dataset. Data collection and sharing for this project was funded by the Alzheimer's Disease Neuroimaging Initiative (ADNI) (National Institutes of Health Grant U01 AG024904). ADNI is funded by the National Institute on Aging, the National Institute of Biomedical Imaging and Bioengineering, and through generous contributions from the following: Alzheimer's Association; Alzheimer's Drug Discovery Foundation; BioClinica, Inc.; Biogen Idec Inc.; Bristol-Myers Squibb Company; Eisai Inc.; Elan Pharmaceuticals, Inc.; Eli Lilly and Company; F. Hoffmann-La Roche Ltd and its affiliated company Genentech, Inc.; GE Healthcare; Innogenetics, N.V.; IXICO Ltd.; Janssen Alzheimer Immunotherapy Research & Development, LLC.; Johnson & Johnson Pharmaceutical Research & Development LLC.; Medpace, Inc.; Merck & Co., Inc.; Meso Scale Diagnostics, LLC.; NeuroRx Research; Novartis Pharmaceuticals Corporation; Pfizer Inc.; Piramal Imaging; Servier; Synarc Inc.; and Takeda Pharmaceutical Company. The Canadian Institutes of Health Research is providing funds to support ADNI clinical sites in Canada. Private sector contributions are facilitated by the Foundation for the National Institutes of Health (www.fnih.org). The grantee organization is the Northern California Institute for Research and Education, and the study is coordinated by the Alzheimer's Disease Cooperative Study at the University of California, San Diego. ADNI data are disseminated by the Laboratory for Neuro Imaging at the University of California, Los Angeles. This research was also supported by NIH grants P30 AG010129 and K01 AG030514.

We thank the IGAP for providing summary results data of their analyses. The

investigators within IGAP contributed to the design and implementation of IGAP and/or provided data but did not participate in analysis or writing of this report. IGAP was made possible by the generous participation of the control subjects, the patients, and their families. The i–Select chips was funded by the French National Foundation on Alzheimer's disease and related disorders. EADI was supported by the LABEX (laboratory of excellence program investment for the future) DISTALZ grant, Inserm, Institut Pasteur de Lille, Université de Lille 2 and the Lille University Hospital. GERAD was supported by the Medical Research Council (Grant n° 503480), Alzheimer's Research UK (Grant n° 503176), the Wellcome Trust (Grant n° 082604/2/07/Z) and German Federal Ministry of Education and Research (BMBF): Competence Network Dementia (CND) grant n° 01GI0102, 01GI0711, 01GI0420. CHARGE was partly supported by the NIH/NIA grant R01 AG033193 and the NIA AG081220 and AGES contract N01–AG–12100, the NHLBI grant R01 HL105756, the Icelandic Heart Association, and the Erasmus Medical Center and Erasmus University. ADGC was supported by the NIH/NIA grants: U01 AG032984, U24 AG021886, U01 AG016976, and the Alzheimer's Association grant ADGC–10–196728.
